# Towards a Computational Framework for Modeling the Impact of Aortic Coarctations Upon Left Ventricular Load

**DOI:** 10.3389/fphys.2018.00538

**Published:** 2018-05-28

**Authors:** Elias Karabelas, Matthias A. F. Gsell, Christoph M. Augustin, Laura Marx, Aurel Neic, Anton J. Prassl, Leonid Goubergrits, Titus Kuehne, Gernot Plank

**Affiliations:** ^1^Computational Cardiology Laboratory, Institute of Biophysics, Medical University of Graz, Graz, Austria; ^2^Shadden Research Group, Department of Mechanical Engineering, University of California, Berkeley, Berkeley, CA, United States; ^3^Department of Congenital Heart Disease/Pediatric Cardiology, German Heart Institute Berlin, Berlin, Germany; ^4^Institute for Imaging Science and Computational Modeling in Cardiovascular Medicine, Charité - University Medicine Berlin, Berlin, Germany

**Keywords:** cardiac mechanics, computational fluid dynamics, finite element model, arbitrary Lagrangian-Eulerian formulation, patient-specific modeling, translational cardiac modeling, total heart function

## Abstract

Computational fluid dynamics (CFD) models of blood flow in the left ventricle (LV) and aorta are important tools for analyzing the mechanistic links between myocardial deformation and flow patterns. Typically, the use of image-based kinematic CFD models prevails in applications such as predicting the acute response to interventions which alter LV afterload conditions. However, such models are limited in their ability to analyze any impacts upon LV load or key biomarkers known to be implicated in driving remodeling processes as LV function is not accounted for in a mechanistic sense. This study addresses these limitations by reporting on progress made toward a novel electro-mechano-fluidic (EMF) model that represents the entire physics of LV electromechanics (EM) based on first principles. A biophysically detailed finite element (FE) model of LV EM was coupled with a FE-based CFD solver for moving domains using an arbitrary Eulerian-Lagrangian (ALE) formulation. Two clinical cases of patients suffering from aortic coarctations (CoA) were built and parameterized based on clinical data under pre-treatment conditions. For one patient case simulations under post-treatment conditions after geometric repair of CoA by a virtual stenting procedure were compared against pre-treatment results. Numerical stability of the approach was demonstrated by analyzing mesh quality and solver performance under the significantly large deformations of the LV blood pool. Further, computational tractability and compatibility with clinical time scales were investigated by performing strong scaling benchmarks up to 1536 compute cores. The overall cost of the entire workflow for building, fitting and executing EMF simulations was comparable to those reported for image-based kinematic models, suggesting that EMF models show potential of evolving into a viable clinical research tool.

## 1. Introduction

CFD models of blood flow in the LV and aorta are important tools for analyzing the mechanistic links between myocardial deformation and flow patterns. Typically, such models are either driven by prescribed flow profiles measured in the LV outflow tract or the aortic root (Goubergrits et al., 2013; Ralovich et al., 2015; Andersson et al., 2017), or by image-based kinematic models (Doenst et al., 2009; Schenkel et al., 2009; Mihalef et al., 2011; Seo et al., 2013; Chnafa et al., 2014; Su et al., 2016) built from segmentation of 4D medical imaging datasets. While such models have proven to be valuable for analyzing the hemodynamic status quo of a patient or for predicting changes in hemodynamics in the aorta secondary to intervention such as aortic valve repair (Kelm et al., 2017) or stenting of a coarctation (Goubergrits et al., 2015), they are inherently limited in their ability to assess cardiac function as the biophysics driving myocardial activation and deformation is not taken into consideration in the model formulation. EMF models that capture the entire physics of a heartbeat based on first principles show promise to overcome this limitation (Crozier et al., 2016a) by rendering feasible the assessment of all essential myocardial parameters, which are known to be key factors driving ventricular remodeling and disease progression. Thus EMF models may offer, in principal, the potential of predicting longer term outcomes beyond changes in the acute response to therapies.

However, due to a number of factors such as the inherent complexity of multiphysics models, the large-scale motion and complex deformation of the myocardial walls as well as the significant computational burden, these models pose substantial methodological challenges. For LV EMF models and similar applications, methods to overcome the problem of large-scale deformations can be roughly classified into two categories: ALE formulations using a moving fluid mesh (Tang et al., 2008, 2010; Nordsletten et al., 2011; Vázquez et al., 2015; de Vecchi et al., 2016) and immersed boundary (IB) methods (Vigmond et al., 2008a; Seo and Mittal, 2013; Choi et al., 2015). While ALE formulations often rely on severe simplifications or automatic remeshing strategies (Long et al., 2013), IB methods are more versatile as the moving wall of the ventricle is not explicitly tracked. However, IBs and all related non-boundary-fitting methods have a reduced accuracy for the solution near the fluid-solid structure interface due to interpolation errors, pose severe challenges on the implementation, and additional degrees of freedom have to be introduced on interface cut elements, which all contributes to significantly higher computational costs (van Loon et al., 2007).

In this study, we report on the progress made toward a novel EMF model of the human LV that is entirely based on first principles and that copes with significantly large defomations, i.e., ejection fractions (EFs) beyond 60%, without requiring remeshing or IB principles. Validated *in silico* models taken from a recent clinical modeling study where a cohort of *in silico* EM LV and aorta models of patients suffering from aortic valve disease (AVD) and/or CoA (Augustin et al., 2016a) were built, served as kinematic driver to a computational model of hemodynamics in the LV cavity and aorta. A hybrid two stage modeling approach was adopted with regard to hemodynamics. First, the afterload imposed by the circulatory system onto the LV was represented by a lumped model of afterload and coupled to an EM model of LV and aorta to compute LV kinematics. Subsequently a full-blown CFD model with moving domain boundaries based on an ALE formulation was *unidirectionally* or *weakly* coupled to the EM model using the kinematics of its endocardial surface as input. We show validation results for two selected clinical CoA cases under pre-treatment conditions and compare pre-treatment and post-treatment simulation results for one patient case in which the CoA was geometrically repaired by a virtual stenting procedure. Further, we demonstrate numerical feasibility of the implemented approach by analyzing changes in mesh quality and its impact upon solver performance under the significantly large deformations of the LV blood pool mesh and also provide strong scaling benchmarking results for a range of 96–1,536 compute cores. The overall cost of the entire workflow for building, fitting and execution of EMF simulations is ≈ 48 h which is comparable to plain image-based kinematic driver models (Mittal et al., 2016).

## 2. Methods

The methodology to develop a coupled model of cardiac and cardiovascular hemodynamics based on an ALE formulation is structured as follows.

- We begin in section 2.1 by describing MRI data acquisition and anatomical FE model generation of the LV and aorta for two patients suffering from CoA.
- Then, a brief summary of all model components is given comprising an electrophysiology (EP) model to drive electrical activation and repolarization (section 2.2.1); an EM model describing passive biomechanics as well as the generation of active stresses (section 2.2.2); afterload models to provide appropriate boundary conditions on the LV endocardium during the ejection phase (section 2.2.3); and a CFD model with moving domain boundaries representing blood flow in the LV and aorta during ejection. The EM and CFD model are weakly coupled in a forward fluid structure interaction (FSI) framework, where the EM model is used as a kinematic driver to move the fluid domain (section 2.3).
- The solution procedure and software implementation details are outlined in section 2.4.
- Finally, procedures implemented for the patient-specific parameterization of the major model components is described in section 2.5.

### 2.1. Clinical data acquisition and model generation

Hemodynamic data of two patients with clinical indication for catheterization due to CoA—all preceding a cardiac magnetic resonance study—were acquired before and after CoA treatment by stent implant, see Table 1. CoA treatment indicators included an echocardiographic measured, peak systolic pressure gradient across the stenotic region of > 20 mmHg and/or arterial hypertension. The study was approved by the institutional research ethics committee following the ethical guidelines of the 1975 Declaration of Helsinki. Written informed consent was obtained from the participants' guardians. Acquired data are summarized in Table 1.

**Table 1:** CoA patient characteristics from MRI and invasive catheter pressure recordings including end-diastolic volume (EDV), end-systolic volume (ESV), stroke volume (SV), ejection fraction (EF), heart rate (HR), cardiac output (CO), diastolic and systolic pressures recorded in the aorta or estimated from cuff measurements (*P*_ao/cuff,dia_ and *P*_ao/cuff, sys_), mean arterial pressure (MAP) computed from pressure recorded invasively in the aorta or estimated from *P*_cuff,dia_ and *P*_cuff,sys_, and aortic valve open pressure *P*_open_ determined from invasive pressure recordings.

	**Sex**	**Age**	**EDV**	**ESV**	**SV**	**EF**	**HR**	**CO**	**$P_{\text{ao/cuff}}^{\text{dia}}$**	**$P_{\text{ao/cuff}}^{\text{sys}}$**	**MAP**	***P*_open_**
			**(ml)**	**(ml)**	**(ml)**	**(%)**	**(bpm)**	**(ml/s)**	**(mmHg)**	**(mmHg)**	**(mmHg)**	**(mmHg)**
*28-Pre*	F	9	88.2	30.6	57.54	65.3	91	87.46	71.1/62	122.7/138	88.3/87.3	71.33
*44-Pre*	M	12	91.7	31.6	60.09	65.5	76	76.31	74.6/120	125.2/154	91.5/131.3	74.78

#### 2.1.1. MRI acquisition and post processing

MR imaging was done with a whole body 1.5 Tesla MR scanner Achieva R 3.2.2.0 using a five-element cardiac phased-array coil (Philips Medical System, Best, Netherlands). Three MRI sequences were used further in our study: (i) flow-sensitive four-dimensional (4D) velocity-encoded magnetic resonance imaging (4D VEC-MRI), (ii) three-dimensional (3D) anatomical imaging of the whole heart (3DWH) during diastasis, and (iii) 4D gapless short axis Cine MRI.

4D VEC-MRI of the thorax was performed using an anisotropic 4D segmented *k*-space phase contrast gradient echo sequence. Retrospective electrocardiographic gating without navigator gating of respiratory motion in order to minimize acquisition time was used. Sequence parameters were: acquired voxel 2.5 × 2.5 × 2.5 mm; reconstructed voxel 1.7 × 1.7 × 2.5 mm; repetition time 3.5 ms; echo time 2.2 ms; flip angle 5°; 25 reconstructed cardiac phases; number of signal averages 1; High velocity encoding (3–6 m/s) in all three directions was used in order to avoid phase wraps in the presence of coarctation and associated secondary flow. Flow measurements were completed with automatic correction of concomitant phase errors. Postprocessing for analysis of flow rates across the aortic valve was carried out with GTFlow 1.6.8 software^1^ (Gyrotools, Zurich, Switzerland).

The 3DWH exemplary sequence parameters were: acquired voxel 0.66 × 0.66 × 3.2 mm; reconstructed voxel 0.66 × 0.66 × 1.6 mm; repetition time 4.0 ms; echo time 2.0 ms; flip angle 90°; and number of signal averages 3.

Short axes Cine imaging data were acquired with sequence parameters: 16 slices, with an acquisition resolution of 0.86 × 0.86 × 6.0 mm, repetition time 4.24 ms, echo time 2.12 ms, flip angle 60° and 25 automatically reconstructed cardiac phases which were used to determine LV volume traces. The non-compact myocardium as well as papillary muscles were counted toward blood pool volume.

MRI based pressure mapping allowing to assess non-invasively the relative pressures in a vessel by solving Pressure Poisson equation (PPE) was done with MevisFlow^2^. Briefly, the PPE can be derived from the Navier–Stokes equations by taking the divergence of the momentum Equation (26), see Gresho and Sani (1987) and Krittian et al. (2012) for more details. For more details we refer to Krittian et al. (2012). The processing and analysis pipeline of the pressure mapping consists of the following four steps.

- Semi-automatic segmentation (labeling) of the aortic domain from 3DWH data generating 3D mask of the aorta.
- Background phase correction and phase-unwrapping of the 4D VEC-MRI data and generation of a sequence of volumetric velocity vector fields.
- Coarse semi-automatic segmentation of the aorta based on magnitude and phase contrast of the 4D VEC-MRI data and registration with 3DWH based mask of the aorta.
- Solving (PPE) at each time step having 4D VEC-MRI data as input. Furthermore, a 5 % mask size reduction is applied in order to avoid numerical inconsistencies close to the vessel wall as suggested earlier (Meier et al., 2010).

Relative pressure maps are represented with zero pressure located at the center of the CoA (narrowest location). 3D mask based on 3DWH data was used due to its better spatial resolution compared to 4D VEC-MRI data. Correction of velocity data (step ii) was done in order to minimize noise and aliasing artifacts originating from multiple sources.

#### 2.1.2. Invasive catheter recordings

During catheterization, pressure was recorded over the cardiac cycle in the ascending aorta and the LV before treatment and repeated in the ascending aorta after an interventional treatment procedure was performed. Pressures were recorded simultaneously at three predefined locations (LV, ascending aorta, and descending aorta) and the femoral artery during catheterization. Patients were sedated by intravenous administration of a bolus of midazolam (0.1–0.2 mg/kg, max. 5 mg), followed by a bolus of propofol (1–2 mg/kg, as needed) and continuous infusion of propofol (1–2 mg/kg, as needed). Pressure measurements were taken with senior cardiologists present. Pigtail catheters (Cordis, Warren, NJ, USA) of 5-6F were connected to pressure transducers (Becton-Dickinson, Franklin Lakes, NJ, USA). Routinely, patients received balloon angioplasty with or without additional placement of a stent in order to treat a given stenosis by removing the narrowing of the vessel and thus the pressure gradient. To reduce duration of catheterization, pressures were measured post-treatment only in the ascending aorta. The Schwarzer hemodynamic analysis system (Schwarzer, Heilsbronn, Germany) was used to amplify, acquire, and analyze pressure signals.

#### 2.1.3. Anatomical FE model generation

Multi-label segmentation of the LV myocardium, LV blood pool, left atrium (LA) and aortic cavities was done at the DHZB using 3DWH data and the ZIB Amira software^3^ (Stalling et al., 2005). The segmentations were smoothed and upsampled to a 0.1 mm isotropic resolution using a variational smoothing method (Crozier et al., 2016a). The resulting high resolution multi-label segmentation was meshed using CGAL^4^ (The CGAL Project, 2017), giving a global mesh $\Omega_{\text{s},\text{total}}^{0}$ consisting of tetrahedral elements. Here, (•)^0^ denotes the mechanical reference configuration at end-diastolic pressure. The mesh was subdivided into various subdomains corresponding to predefined labels which are summarized in Equation (3). We write

(1) $$\Omega_{\text{s},\text{total}}^{0}=\underset{i\in I}{\cup }\Omega_{\text{s},i}^{0},$$

with the index set

**Figure 1:**
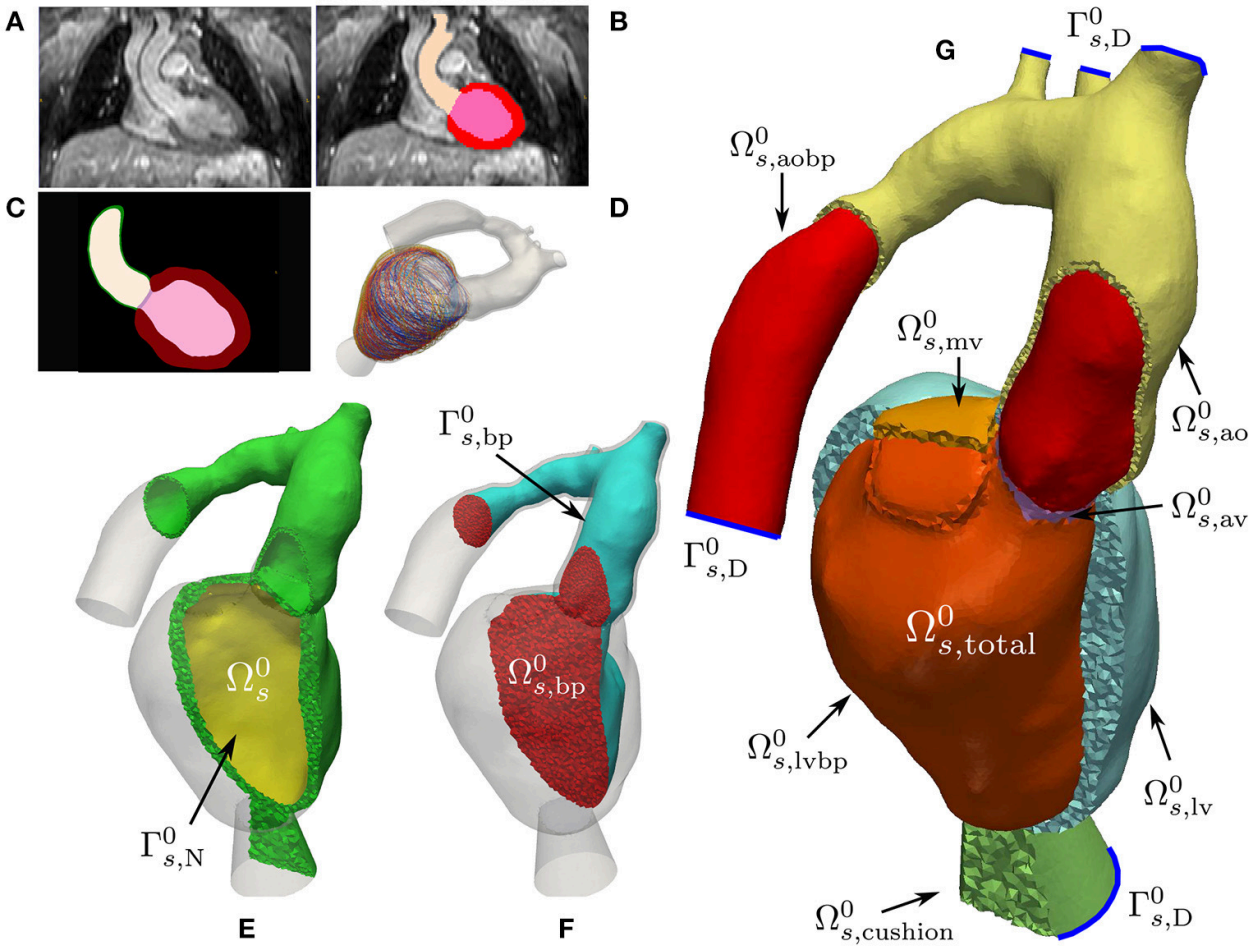
Mechanics model generation: Starting from a patient specific MRI scan **(A)** a segmentation was performed **(B)** which was then upsampled and smoothed **(C)**. Myocardial fibers were generated in the tissue according to Bayer et al. (2012) **(D)**. A labeled FE geometry $\Omega_{\text{s},\text{total}}^{0}$ including the blood pool was generated **(G)**. The geometry has been sliced to reveal the blood pool and valves and has been color coded according to the labels defined in Equation (3). Boundaries $\Gamma_{\text{s},\text{D}}^{0}$ used for prescribing homogeneous Dirichlet boundary conditions are sketched as blue curves. From this mesh the EM submesh $\Omega_{\text{s}}^{0}$ **(E)** and the unsmoothed blood pool **(F)** were extracted. Boundary $\Gamma_{\text{s},\text{N}}^{0}$ was used to prescribe pressure boundary conditions inside the LV and $\Gamma_{\text{s},\text{bp}}^{0}$ is the surface of the blood pool.

(2) I:={lv,ao,cushion,av,mv,lvbp,aobp},

see Figures 1E–G for illustration. The elements in the index set are abbreviations for the following labels

(3) $$\begin{array}{l} \text{ lv }↔\text{Myocardium},\\ \text{ ao }↔\text{Aortic wall},\\ \text{cushion }↔\text{Elastic cushion},\\ \text{ av }↔\text{Aortic valve},\\ \text{ mv }↔\text{Mitral valve},\\ \text{ lvbp }↔\text{Left ventricular bloodpool},\\ \text{ aobp }↔\text{Aortic bloodpool}. \end{array}$$

With this, we define the following submeshes

(4) $$\Omega_{\text{s}}^{0}\;:\text{​​}=\Omega_{\text{s},\text{total}}^{0}\backslash \left(\Omega_{\text{s},\text{lbvp}}^{0}\cup \Omega_{\text{s},\text{aobp}}^{0}\right),$$

(5) $$\Omega_{\text{s},\text{bp}}^{0}=\;{\tilde{\Omega }}_{f}^{0}:\text{​​}=\Omega_{\text{s},\text{av}}^{0}\cup \Omega_{\text{s},\text{lvbp}}^{0}\cup \Omega_{\text{s},\text{aobp}}^{0},$$

where $\Omega_{\text{s}}^{0}$ is the solid domain and $\Omega_{\text{s},\text{bp}}^{0}$ is the unsmoothed blood pool domain used for extracting a smoothed CFD mesh, see Figures 1E,F. For later use, we define the following surfaces

(6) $$\Gamma_{\text{s},\text{N}}^{0}\;:\text{​​}=\partial \left(\left(\Omega_{s,\text{lv}}^{0}\cup \Omega_{\text{s},\text{av}}^{0}\cup \Omega_{\text{s},\text{mv}}^{0}\right)\cap \Omega_{\text{s},\text{lvbp}}^{0}\right),$$

(7) $$\Gamma_{\text{s},\text{H}}^{0}\;:\text{​​}=\partial \Omega_{\text{s}}^{0}\backslash \left(\Gamma_{\text{s},\text{N}}^{0}\cup \Gamma_{\text{s},\text{D}}^{0}\right),$$

(8) $$\Gamma_{\text{s},\text{bp}}^{0}\;:\text{​​}=\partial \Omega_{\text{s},\text{bp}}^{0}\backslash \Gamma_{\text{s},\text{D}}^{0},$$

where $\Gamma_{\text{s},\text{D}}^{0}$ denote the cutoff faces as indicated by blue lines in Figure 1; $\Gamma_{\text{s},\text{N}}^{0}$ are surfaces subject to pressure; and $\Gamma_{\text{s},\text{H}}^{0}$ are surfaces with homogeneous Neumann boundary conditions. In order to avoid numerical difficulties with non-smooth, jagged boundaries, the surface of the mechanical blood pool domain $\Gamma_{\text{s},\text{bp}}^{0}$ was extracted and smoothed using the VMTK toolbox^5^ (Antiga et al., 2008). The smoothed surface, $\Gamma_{\text{f},\text{wall}}^{0}$, was used to define the boundary of the fluid domain reference configuration, $\Omega_{\text{f}}^{0}$, for volumetric FE meshing using ANSYS ICEM CFD^6^. Refined boundary layers were included in this process to better resolve sharp gradients in the vicinity of $\Gamma_{\text{f},\text{wall}}^{0}$ occurring during simulation of hemodynamics. The various processing stages for building EM and CFD models are illustrated in Figures 1, **4**, respectively.

### 2.2. Electromechanical model

#### 2.2.1. Electrophysiology of the LV

A recently developed reaction-eikonal (R-E) model (Neic et al., 2017) was employed to generate electrical activation sequences which serve as a trigger for active stress generation in cardiac tissue. The hybrid R-E model combines a standard reaction-diffusion (R-D) model based on the monodomain equation with an eikonal model. Briefly, the eikonal equation is given as

(9) $$\{\begin{array}{c} \sqrt{\nabla_{\text{X}}t_{\text{a}}^{⊤}\text{V}\nabla_{\text{X}}t_{\text{a}}}=1\text{ in }\Omega_{\text{s},\text{lv}}^{0},\\ \;t_{\text{a}}=t_{0}\text{ on }\Gamma_{\text{s},*}^{0}, \end{array}$$

where (∇_**X**_) is the gradient with respect to the end-diastolic reference configuration $\Omega_{\text{s},\text{lv}}^{0}$; *t*_a_ is a positive function describing the wavefront arrival time at location $\text{X}\in \Omega_{\text{s},\text{lv}}^{0}$; and *t*_0_ are initial activations at locations $\Gamma_{\text{s},*}^{0}\subseteq \Gamma_{\text{s},\text{N}}^{0}$. The symmetric positive definite 3 × 3 tensor **V**(**X**) holds the squared velocities (*v*_f_(**X**), *v*_s_(**X**), *v*_n_(**X**)) associated to the tissue's eigenaxes, referred to as fiber, **f**_0_, sheet, **s**_0_, and sheet normal, **n**_0_, orientations. The arrival time function *t*_a_(**X**) was subsequently used in a modified monodomain R-D model given as

(10) $$\beta C_{\text{m}}\frac{\partial V_{\text{m}}}{\partial t}=\nabla_{\text{X}}\cdot \;\sigma_{\text{i}}\nabla_{\text{X}}V_{\text{m}}+I_{\text{foot}}-\beta I_{\text{ion}},$$

where an arrival time dependent foot current, *I*_foot_(*t*_a_), was added which is designed to mimic subthreshold electrotonic currents to produce a physiological foot of the action potential. The key advantage of the R-E model is its ability to compute activation sequences at much coarser spatial resolutions that are not afflicted by the spatial undersampling artifacts leading to conduction slowing or even numerical conduction block as it is observed in standard R-D models. Ventricular EP was represented by the tenTusscher–Noble–Noble–Panfilov model of the human ventricular myocyte (ten Tusscher et al., 2004). As indicated in Equations (9, 10), activation sequences and electrical source distribution in the LV were computed in its end-diastolic configuration $\Omega_{\text{s},\text{lv}}^{0}$, that is, any effects of deformation upon electrotonic currents remained unaccounted for.

#### 2.2.2. Active and passive mechanics in the LV and aorta

The deformation of the heart is governed by imposed external loads such as pressure in the cavities or from surrounding tissue and active stresses intrinsically generated during contraction. Tissue properties of the LV myocardium and the aorta are characterized as a hyperelastic, nearly incompressible, anisotropic material with a non-linear stress-strain relationship. Mechanical deformation was described by Cauchy's equation of motion under stationary equilibrium assumptions leading to a quasi-static boundary value problem

(11) $$-\nabla_{\text{X}}\cdot \text{FS}({\text{d}}_{\text{s}},t)=0\text{ in }\Omega_{\text{s}}^{0},$$

for *t* ∈ [0, *T*], where **d**_s_ is the unknown displacement; **F** is the deformation gradient; **S** is the second Piola–Kirchhoff stress tensor; and (∇_**X**_ ·) denotes the divergence operator in the Lagrange reference configuration. Homogeneous Dirichlet boundary conditions

(12) $${\text{d}}_{\text{s}}=0\text{ on }\Gamma_{\text{s},\text{D}}^{0},$$

homogeneous Neumann boundary conditions

(13) $$\begin{array}{lll} FS({\vec{d}}_{\text{s}},t)=\vec{0} & \; & \text{on }\Gamma_{\text{s},\text{H}}^{0}, \end{array}$$

and inhomogeneous Neumann boundary conditions

(14) $$\text{FS}({\text{d}}_{\text{s}},t){\text{n}}_{\text{s,0}}=p(t)J{\text{F}}^{-⊤}({\text{d}}_{\text{s}},t){\text{n}}_{\text{s},\text{0}}\text{ on }\Gamma_{\text{s},\text{N}}^{0}$$

were imposed, where **n**_s,0_ is the outward unit normal vector; *p*(*t*) is the pressure; and *J* = det **F**. For sake of clarity, boundary conditions are illustrated in Figure 1C.

The total stress **S** was additively decomposed according to

(15) $$\text{S}={\text{S}}_{\text{pas}}+{\text{ S}}_{\text{act}},$$

where **S**_pas_ and **S**_act_ refer to the passive and active stresses, respectively. Passive stresses were modeled based on the constitutive equation

(16) $${\text{S}}_{\text{pas}}=2\frac{\partial \Psi (\text{C})}{\partial \text{C}}$$

given a hyper-elastic strain-energy function Ψ and the right Cauchy–Green strain tensor **C** = **F**^⊤^**F**. Two different strain-energy functions were used for characterizing passive mechanical behavior in the LV and the aorta. In the LV, where the underlying mesh $\Omega_{\text{s},\text{lv}}^{0}$ and fiber orientations (**f**_0_, **s**_0_, **n**_0_) are the same as for the EP model, section 2.2.1, the transversely isotropic constitutive relation

(17) $$\Psi_{\text{Guc}}(\text{C})=\frac{\kappa }{2}{\left(\log J\right)}^{2}+\frac{C_{\text{Guc}}}{2}\left[\exp(Q)-1\right].$$

by Guccione et al. (1995) was employed. Here, the term in the exponent is

(18) $$\begin{array}{l} Q\;=b_{\text{f}}{\left({\text{f}}_{0}\cdot \bar{\text{E}}{\text{f}}_{0}\right)}^{2}+b_{\text{t}}\;\left[{\left({\text{s}}_{0}\cdot {\bar{\text{E}}\text{s}}_{0}\right)}^{2}+{\left({\text{n}}_{0}\cdot {\bar{\text{E}}\text{n}}_{0}\right)}^{2}+2{\left({\text{s}}_{0}\cdot {\bar{\text{E}}\text{n}}_{0}\right)}^{2}\right]\\ \;+2b_{\text{fs}}\left[{\left({\text{f}}_{0}\cdot {\bar{\text{E}}\text{s}}_{0}\right)}^{2}+{\left({\text{f}}_{0}\cdot {\bar{\text{E}}\text{n}}_{0}\right)}^{2}\right] \end{array}$$

and $\bar{\text{E}}=\frac{1}{2}\left(\bar{\text{C}}-\text{I}\right)$ is the modified isochoric Green–Lagrange strain tensor, where $\bar{\text{C}}:=J^{-2/3\text{C}}$. Default values of *b*_f_ = 18.48, *b*_t_ = 3.58, and *b*_fs_ = 1.627 were used. The parameter *C*_Guc_ was varied for the different cases, see Table 2. In the aorta $\Omega_{\text{s},\text{ao}}^{0}$, unlike in previous studies (Augustin et al., 2014), we refrained from assigning fiber structures, since our efforts were primarily focused on modeling the biomechanics of the LV and, to a lesser degree, the aorta. Thus, in absence of information on structural anisotropy, an isotropic model due to Demiray (1972) was used

(19) $$\Psi_{\text{Dem}}(\text{C}):=\frac{\kappa }{2}{\left(\log J\right)}^{2}+\frac{a}{2b}\left\{\exp\left[b(\text{tr}(\bar{\text{C}})-3)\right]-1\right\}.$$

The parameter $\tilde{C}=\frac{a}{2b}$ was chosen such that $\tilde{C}$ = 3,000 kPa in the aorta, $\tilde{C}$ = 30,000 kPa for valves, and $\tilde{C}=300\;kPa$ for the elastic cushion. The bulk modulus κ, which serves as a penalty parameter to enforce nearly incompressible material behavior, was chosen as κ = 650 kPa in both Equations (17, 19). For the elastic cushion a value of κ = 100 kPa was used.

**Table 2:** Fitted parameters for EM Model.

	**EM fitting**								
	***S*_peak_**	***t*_dur_**	**τ_c0_**	**τ_r_**	***t*_emd_**	***C*_Guc_**	***R***	***Z***	***C***
	**(kPa)**	**(ms)**	**−**	**−**	**(ms)**	**(kPa)**	**(kPa ms/ml)**	**(kPa ms/ml)**	**(ml/kPa)**
*28-Pre*	60.0	380	30.0	30.0	15.0	0.48	170.65	12.00	6.75
*28-Post*	55.0	380	30.0	30.0	15.0	0.48	170.65	12.00	6.75
*44-Pre*	90.0	400	50.0	50.0	15.0	0.48	166.65	13.33	7.42

A simplified phenomenological contractile model was used to represent active stress generation (Niederer S. A. et al., 2011). Owing to its small number of parameters and its direct relation to clinically measurable quantities such as peak pressure, *p*_lv_, and the maximum rate of rise of pressure, d*p*_lv_/d*t*_max_, this model is fairly easy to fit and thus very suitable for being used in clinical EM modeling studies. Briefly, the active stress transient is given by

(20) $$\begin{array}{l} S_{\text{a}}(t,\lambda )=S_{\text{peak}}\phi (\lambda )\tanh^{2}\left(\frac{t_{\text{s}}}{\tau_{\text{c}}}\right)\tanh^{2}\left(\frac{t_{\text{dur}}-t_{\text{s}}}{\tau_{\text{r}}}\right),\\ \text{ for }0<t_{\text{s}}<t_{\text{dur}}, \end{array}$$

with

(21) $$\phi =\tanh(\text{ld}(\lambda -\lambda_{0})),\;\tau_{\text{c}}=\tau_{{\text{c}}_{0}}\;+\;{\text{ld}}_{\text{up}}(1-\phi ),\;t_{\text{s}}=t\;-\;t_{\text{a}}\;-\;t_{\text{emd}}$$

and *t*_s_ is the onset of contraction; ϕ(λ) is a non-linear length-dependent function in which λ is the fiber stretch and λ_0_ is the lower limit of fiber stretch below which no further active tension is generated; *t*_a_ is the local activation time from Equation (9); *t*_emd_ is the EM delay between the onsets of electrical depolarization and active stress generation; *S*_peak_ is the peak isometric tension; *t*_dur_ is the duration of active stress transient; τ_c_ is time constant of contraction; τ_c_0__ is the baseline time constant of contraction; ld_up_ is the length-dependence of τ_c_; τ_r_ is the time constant of relaxation; and ld is the degree of length dependence. Thus, active stresses in this simplified model are only length-dependent, but dependence on fiber velocity, $\dot{\lambda }$, is ignored. Unlinke in previous studies (Niederer S. A. et al., 2011) we set the nonlinear length-dependent function ϕ(λ) = 1 for the whole simulation. The active stress tensor in the reference configuration $\Omega_{\text{s},\text{lv}}^{0}$ induced in fiber direction **f**_0_ is defined as

(22) $${\text{S}}_{\text{a}}=S_{\text{a}}{\left({\text{f}}_{0}\cdot {\text{Cf}}_{0}\right)}^{-1}{\text{f}}_{0}⊗{\text{f}}_{0},$$

with *S*_*a*_ defined in Equation (20). This active stress involves a scaling by $\lambda^{2}={\text{f}}_{0}\cdot \text{C}{\text{f}}_{0}$, see Pathmanathan and Whiteley (2009) for details.

#### 2.2.3. Mechanical and hemodynamic afterload models

**Figure 2:**
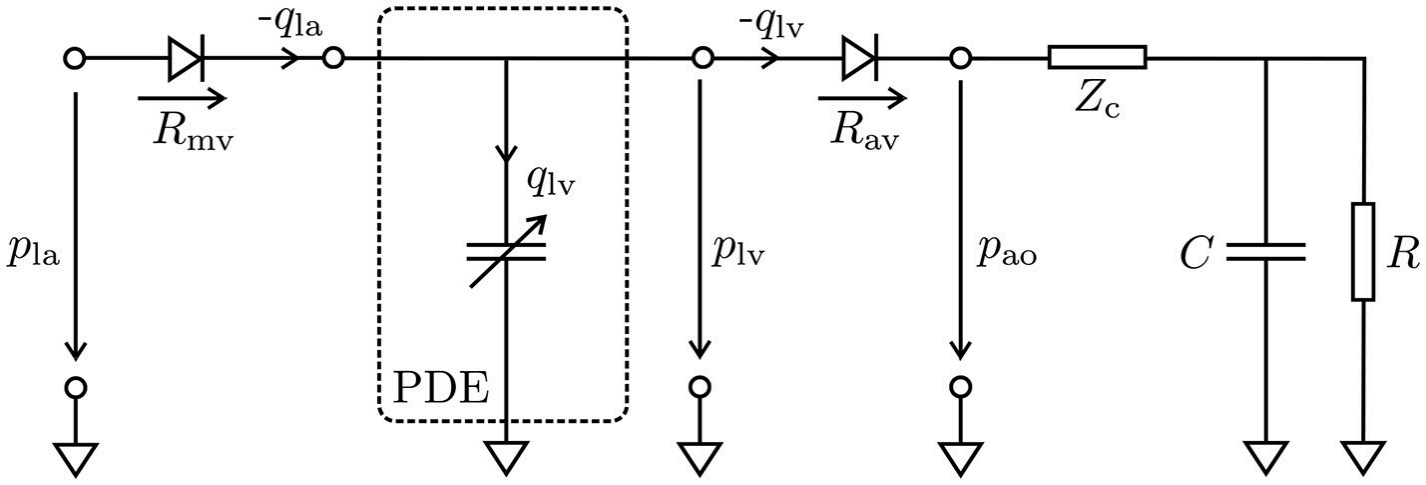
Lumped circuit representation of the coupled EM PDE model of the LV with the cardiovascular system. The time-varying compliance of the LV is represented as a PDE model which was coupled through the aortic valve (*R*_av_) to a 3-element Windkessel model representing aortic impedance, *Z*_c_, and peripheral arterial compliance, *C*, and resistance, *R*, during ejection, and through the mitral valve (*R*_mv_) to a constant pressure *p*_la_ in the left atrium during filling. Negative flows −*q*_la_ and −*q*_lv_ mean the respective cavity is ejecting, while positive flow means cavity is being filled.

Hydrostatic pressures in the LV, *p*_lv_, and the proximal aorta, *p*_ao_, were modeled using a 3-element Windkessel model (Westerhof et al., 1971), and the system of PDEs (11) was linked to this lumped model of the arterial system, see Figure 2. The models were coupled by a diode (aortic valve) which opens at the end of the isovolumetric contraction (IVC) phase when the pressure in the LV cavity, *p*_lv_, exceeds the pressure in the proximal aorta, *p*_ao_, and closes at the end of ejection when *p*_lv_ drops below *p*_ao_ and the flow *q*_lv_ starts to reverse. In its open state the aortic valve was modeled as a linear resistor, *R*_av_, in series with the characteristic impedance of the aorta, *Z*_c_. During ejection, the pressure in the LV was then computed by the Windkessel equation

(23) $$\frac{\text{d}p_{\text{lv}}}{\text{d}t}=\frac{1}{C}\left(1+\frac{Z_{\text{c}}\;+\;R_{\text{av}}}{R}\right)q_{\text{lv}}\;+\;(Z_{\text{c}}\;+\;R_{\text{av}})\frac{\text{d}q_{\text{lv}}}{\text{d}t}\;-\;\frac{1}{RC}p_{\text{lv}},$$

which predicts the rate of change of pressure in the LV as a function of flow *q*_lv_ out of the LV into the aorta. The resistor *R* represents peripheral arterial resistance placed in parallel with a capacitor *C*, representing vascular compliance.

A similar form of Equation (23) was also used to estimate the pressure in the aorta, *p*_ao_. In this case, there is no additional resistance due to an outlet valve and hence *R*_av_ is omitted. Balancing of the PDE (11) and the ODE (23) was achieved by recasting Equation (11) as a saddle point problem, see Gurev et al. (2015) and Hirschvogel et al. (2017).

For CFD simulations, hydrostatic pressures at artificial aortic fluid outlets, were modeled using a similar 3-element Windkessel model as in Equation (23) that was rewritten in the form of the following differential algebraic equations for outlet *i*

(24) $$C_{i}\frac{\text{d}p_{\text{d},i}}{\text{d}t}+\frac{p_{\text{d},i}}{R_{i}}\;=q_{i},$$

(25) $$p_{\text{wk},i}\;=Z_{i}q_{i}+p_{\text{d},i},$$

see Fouchet-Incaux (2014) and Bertoglio et al. (2017) for more details. During ejection the Windkessel pressure *p*_wk_ at an outlet was then applied as an outflow boundary condition for the fluid flow model, see section 2.5.5. In Equations (24, 25), *C*_*i*_ represents compliance, *Z*_*i*_ impedence, and *R*_*i*_ resistance of the peripheral arteries for the respective aortic outlet and *q*_*i*_ denotes the flux through this outlet. Fitting of the parameters involved will be discussed in section 2.5.5.

### 2.3. Fluid flow model

Human blood in larger vessels such as the LV or the aorta complies with the assumptions of an incompressible, isothermal, Newtonian and single-phase liquid (Nichols et al., 2011). Let $\Omega_{\text{f}}⊊{\mathbb{R}}^{3}$ denote the fluid domain, then the evolution of flow is governed by the incompressible Navier–Stokes equations

(26) $$\begin{array}{ll} \rho_{\text{f}}(\frac{\partial }{\partial t}u_{\text{f}}+u_{\text{f}}\cdot \nabla_{x}u_{\text{f}})-\nabla_{x}\cdot \sigma_{\text{f}}(u_{\text{f}},p_{\text{f}})=0 & \text{in }\Omega_{\text{f}} \end{array},$$

(27) $$\begin{array}{ll} \nabla_{x}\cdot u_{\text{f}}=0 & \text{in } \end{array}\Omega_{\text{f}},$$

(28) $$u_{\text{f}}=0\text{ on }\Gamma_{\text{noslip}},$$

(29) $$u_{\text{f}}=g_{\text{f}}\text{ on }\Gamma_{\text{inflow}}\text{,}$$

(30) $$\begin{array}{ll} \sigma_{\text{f}}n_{\text{f}}-\rho_{\text{f}}\beta \left(u_{\text{f}}\cdot n_{\text{f}}\right)\_u_{\text{f}}=h_{\text{f}} & \text{on }\Gamma_{\text{outflow}} \end{array},$$

(31) $${u_{\text{f}}|}_{t=0}=u_{0},$$

where **u**_f_ denotes fluid velocity in m/s; *p*_f_ is fluid pressure in Pa; ρ_f_ is the density of blood, given as 1.060 kg/m^3^; **σ**_f_ is the fluid stress tensor in, Pa, defined as $-p_{\text{f}}\text{I}+\mu_{\text{f}}\left(\nabla_{\text{x}}{\text{u}}_{\text{f}}+\nabla_{\text{x}}{\text{u}}_{\text{f}}^{⊤}\right)$, with dynamic viscosity of blood μ_f_ given as 0.004 Pa s; **g**_f_, in m/s is a velocity inlet; *p*_wk_, in Pa, is the Windkessel pressure solution to Equations (24, 25); **u**_0_, in m/s, refers to the initial condition; **n**_f_ is the outward unit normal of the fluid domain; and (∇_**x**_) is the gradient and (∇_**x**_·) is the divergence operator in the fluid domain Ω_f_. The sets Γ_noslip_, Γ_inflow_, and Γ_outflow_ denote the complementary subsets of Γ_f_: = ∂Ω_f_ and we assume that |Γ_outflow_| > 0. Note that Equation (29) is given only for the sake of completeness but was not used in this study, as the inflow of blood into the aorta is driven by the motion of the LV thus avoiding the need for prescribing an inflow profile as it is necessary in models which consider the aorta in isolation. For *p*_wk_ ≡ 0, boundary condition Equation (30) is referred to as *directional do-nothing boundary condition*, see Esmaily Moghadam et al. (2011) and Braack et al. (2014), and the term

(32) $$\left(u_{\text{f}}\cdot n_{\text{f}}\right)\_:=\frac{1}{2}\left(u_{\text{f}}\cdot n_{\text{f}}-|u_{\text{f}}\cdot n_{\text{f}}\text{|}\right)$$

is added for backflow stabilization. A value of $\beta >\frac{1}{2}$ was assumed to guarantee stability of the system. However, in practical applications values of $\beta \leq \frac{1}{2}$ were also used without causing numerical issues, see Esmaily Moghadam et al. (2011). In presence of multiple outlets outflow boundary conditions as given in Equation (30) were prescribed at each of the outlets.

#### 2.3.1. Extension to moving geometries

For time-dependent fluid domains, i.e., $\Omega_{\text{f}}=\Omega_{\text{f}}^{t}$, Equations (26–31) need to be modified to account for the domain movement. This requires the linking of the equations governing fluid dynamics—posed in an Eulerian coordinate frame—with the structural mechanics equations—posed in a Lagrangian reference frame. This is achieved by using the ALE formulation which combines both Lagrangian and Eulerian formulation in a generalized description, see Bazilevs et al. (2013, section 1.3) and Hirt et al. (1974). Similar to structural mechanics, a reference fluid configuration $\Omega_{\text{f}}^{0}⊊{\mathbb{R}}^{3}$ is used which we identify with the mesh been generated at end-diastolic state, see section 2.1.3. The coordinate system of the Eulerian frame is denoted by **x** and the reference coordinate system is denoted by **X**. Their relation is given by the ALE mapping **x** = **X** + **d**_f_(*t*, **X**). Here, **d**_f_(*t*, **X**) refers to an arbitrary, not necessarily physical, displacement of points to track the deformation of the fluid domain. Using this ALE mapping the time-dependent moving fluid domain is represented as

(33) $$\Omega_{\text{f}}^{t}:\text{​​}=\left\{x:x=X+d_{\text{f}}(t,X),\forall X\in \Omega_{\text{f}}^{0}\right\}.$$

Further, we define the fluid domain velocity **w**_f_ as

(34) $$w_{\text{f}}:\text{​​}=\frac{\partial }{\partial t}\;{\text{d}}_{\text{f}}{|}_{\text{X}},$$

where $\frac{\partial }{\partial t}\left(\cdot \right){}_{|\text{X}}$ is the derivative with respect to *t* with **X** being fixed, and the moving interface between fluid and solid domain as

(35) $$\Gamma_{\text{f},\text{mov}}^{t}:\text{​​}=\partial \Omega_{\text{f}}^{t}\;\backslash \overset{n_{\text{outlets}}}{\underset{i=1}{\cup }}\Gamma_{\text{f},\text{outflow},i}^{t},$$

where $\Gamma_{\text{f},\text{outflow},i}^{t}$ are the individual aortic outlets. The fluid displacement at this point remains unknown and will be specified in section 2.3.3. Combining these concepts, an ALE description of the Navier–Stokes equations can be derived, see e.g., Bazilevs et al. (2013) and Förster et al. (2006),

(36) $$\rho_{\text{f}}\left(\frac{\partial }{\partial t}u_{\text{f}}{|}_{X}+\left(u_{\text{f}}-w_{\text{f}}\right)\cdot \nabla_{x}u_{\text{f}}\right)-\nabla_{x}\cdot \;\sigma_{\text{f}}(u_{\text{f}},p_{\text{f}})=0\text{ on }\Omega_{\text{f}}^{t},$$

(37) $$\nabla_{x}\cdot \;u_{\text{f}}=0\text{ on }\Omega_{\text{f}}^{t},$$

(38) $$u_{\text{f}}=\;g_{\text{mov}}\text{ on }\Gamma_{\text{f},\text{mov}}^{t},$$

(39) $$\sigma_{\text{f}}(u_{\text{f}},p_{\text{f}})n_{\text{f}}-\rho_{\text{f}}\beta ((u_{\text{f}}-w_{\text{f}})\cdot \;n_{\text{f}})\_u_{\text{f}}=-p_{\text{wk},\text{i}}n_{\text{f}}\text{ on each }\Gamma_{\text{f},\text{outflow},i}^{t},$$

(40) $$u_{\text{f}}{|}_{t=0}\;=u_{0}\text{ in }\Omega_{\text{f}}^{0}.$$

Along $\Gamma_{\text{f},\text{mov}}^{t}$ we imposed equality between fluid velocity and the velocity of the moving surfaces. Boundary condition (Equation 39) is the ALE equivalent of the outflow stabilization in Equation (30), see Bazilevs et al. (2013, section 8.4.2.3). Details on how domain movement and velocity were chosen in our application will be discussed later in sections 2.3.3 and 2.5.5.

#### 2.3.2. Variational formulation of the navier–stokes equations

Following Bazilevs et al. (2007), Bazilevs et al. (2013), and Pauli and Behr (2017), the discrete variational formulation of the ALE Equations (36)–(40) can be stated in the following abstract form: find ${\text{u}}_{\text{f}}^{\text{h}}\in {\left[S_{\text{h},\text{g}}^{1}\left(T_{\text{N}}\right)\right]}^{3},p_{\text{f}}^{\text{h}}\in S_{\text{h}}^{1}\left(T_{\text{N}}\right)$ such that for all ${\text{v}}^{\text{h}}\in {\left[S_{h,\text{0}}^{1}\left(T_{\text{N}}\right)\right]}^{3}$ and for all $q^{\text{h}}\in S_{\text{h}}^{1}\left(T_{\text{N}}\right)$

(41) $$A_{\text{NS}}(v^{\text{h}},q^{\text{h}};u_{\text{f}}^{\text{h}},p_{\text{f}}^{\text{h}})+S_{\text{VMS}}(v^{\text{h}},q^{\text{h}};u_{\text{f}}^{\text{h}},p_{\text{f}}^{\text{h}})=F_{\text{NS}}(v^{\text{h}}),$$

with the classical bilinear form of the Navier–Stokes equations

(42) $$\begin{array}{ll} A_{\text{NS}}(v^{\text{h}},q^{\text{h}};u_{\text{f}}^{\text{h}},p_{\text{f}}^{\text{h}}):\text{​​}= & \rho_{\text{f}}\int_{\Omega_{\text{f}}^{t}}v^{\text{h}}\cdot \left(\frac{\partial }{\partial t}u_{\text{f}}^{\text{h}}+\left(u_{\text{f}}^{\text{h}}-w_{\text{f}}^{\text{h}}\right)\cdot \nabla_{x}u_{\text{f}}^{\text{h}}\right)\text{d}x\\ \; & +\int_{\Omega_{\text{f}}^{t}}\varepsilon (v^{\text{h}}):\sigma_{\text{f}}(u_{\text{f}}^{\text{h}},p_{\text{f}}^{\text{h}})\text{d}x\\ \; & +\int_{\Omega_{\text{f}}^{t}}q^{\text{h}}\nabla_{x}\cdot \;u_{\text{f}}^{\text{h}}\text{ d}x-\rho_{\text{f}}\beta \sum_{i=1}^{n_{\text{outlets}}}\;\\ \; & \int_{\Gamma_{\text{f},\text{outflow},i}^{t}}\text{​}\text{​​​​​}((u_{\text{f}}^{\text{h}}-\;w_{\text{f}}^{\text{h}})\cdot n_{\text{f}})\_v^{\text{h}}\cdot u_{\text{f}}^{\text{h}}{\text{d}}_{s_{x}}, \end{array}$$

the bilinear form *S*_VMS_, which is explained later in Equation (45), and the right-hand side contribution

(43) $$F_{\text{NS}}(v^{\text{h}}):\text{​​}=-\sum_{i=1}^{n_{\text{outlets}}}p_{\text{wk},\text{i}}\int_{\Gamma_{\text{f},\text{outflow},i}^{t}}\text{​}\text{​​​​​}v^{\text{h}}\cdot \;n_{\text{f}}{\text{d}}_{s_{x}}.$$

In Equation (42), **ε** is the strain-rate tensor and ${\text{w}}_{\text{f}}^{\text{h}}$ is the discrete counterpart of the fluid domain velocity **w**_f_, i.e.,

(44) $$w_{\text{f}}^{\text{h}}(t^{n+1},X)=\frac{d_{\text{f}}(t^{n+1},X)-d_{\text{f}}(t^{n},X)}{\Delta t}.$$

The FE function space $S_{\text{h},*}^{1}\left(T_{\text{N}}\right)$ is the conformal trial space of piecewise linear, globally continuous basis functions over a decomposition $T_{\text{N}}$ of $\Omega_{\text{f}}^{t}$ into *N* simplicial elements constrained by **v**^h^ = * on essential boundaries. The FE function space $S_{\text{h}}^{1}\left(T_{\text{N}}\right)$ denotes the same space without constraints. For further details we refer to Brenner and Scott (2007) and Steinbach (2007).

From a mathematical point of view, the Navier–Stokes equation can be seen as a multidimensional convection–diffusion equation with pressure acting as a Lagrangian multiplier of the incompressibility constraint. In the common case where velocity and pressure are retained as unknowns, as above, the Ladyzhenskaya–Babuška–Brezzi (LBB) condition has to be satisfied by the velocity and pressure spaces (Donea and Huerta, 2003). A violation of the LBB condition may lead to pressure oscillations. Stabilization techniques allowing the circumvention of the LBB condition exist and have been extensively studied (see for example Hughes et al., 1986; Franca and Hughes, 1988; Douglas and Wang, 1989; Bochev et al., 2006). However, with increasing Reynolds number the Navier–Stokes equations become convection dominated. This requires increasingly finer mesh resolutions to accurately resolve finer flow details which, eventually, renders numerical solution in this form computationally intractable. As a remedy, one can resort to using turbulence models. In particular, in this study the *residual based variational multiscale turbulence model* (RBVMS), see Hughes (1995), Bazilevs et al. (2007), Bazilevs et al. (2013), and Pauli and Behr (2017) was employed which acts as a stabilization and a turbulence model. The underlying main idea is to split the unknown solution into resolvable (coarse) and unresolvable (fine) scales by the FE approximation, where the finer scale details are taken into account based on element residuals. For details on the derivation we refer to elsewhere (Bazilevs et al., 2007). The term *S*_VMS_ in Equation (41) denotes the bilinear form of the RBVMS formulation and reads as

(45) $$\begin{array}{l} S_{\text{VMS}}(v^{\text{h}},q^{\text{h}};u_{\text{f}}^{\text{h}},p_{\text{f}}^{\text{h}}):\text{​​}=\\ \frac{1}{\rho_{\text{f}}}\sum_{l=1}^{n_{\text{el}}}\int_{\tau_{\ell }}\tau_{\text{MOM}}\left(\rho_{\text{f}}\left(u_{\text{f}}^{\text{h}}-w_{\text{f}}^{\text{h}}\right)\cdot \nabla_{x}v^{\text{h}}+q^{\text{h}}\right)\cdot r_{\text{MOM}}(u_{\text{f}}^{\text{h}},p_{\text{f}}^{\text{h}})\text{d}x\\ +\sum_{l=1}^{n_{\text{el}}}\int_{\tau_{\ell }}\tau_{\text{CONT}}\nabla_{x}\cdot \;v^{\text{h}}\nabla_{x}\cdot \;u_{\text{f}}^{\text{h}}\text{d}x\\ -\sum_{l=1}^{n_{\text{el}}}\int_{\tau_{\ell }}\tau_{\text{MOM}}v^{\text{h}}\cdot \left(\nabla_{x}\;u_{\text{f}}^{\text{h}}r_{\text{MOM}}(u_{\text{f}}^{\text{h}},p_{\text{f}}^{\text{h}})\right)\text{d}x\\ -\frac{1}{\rho_{\text{f}}}\sum_{l=1}^{n_{\text{el}}}\int_{\tau_{\ell }}\tau_{\text{MOM}}^{2}\varepsilon (v^{\text{h}}):\;(r_{\text{MOM}}(u_{\text{f}}^{\text{h}},p_{\text{f}}^{\text{h}})⊗r_{\text{MOM}}(u_{\text{f}}^{\text{h}},p_{\text{f}}^{\text{h}}))\text{dx}, \end{array}$$

where the vector **r**_MOM_ is defined as

(46) $$r_{\text{MOM}}(u_{\text{f}}^{\text{h}},p_{\text{f}}^{\text{h}}):\text{​​}=\rho_{\text{f}}\left(\frac{\partial }{\partial t}u_{\text{f}}^{\text{h}}+\left(u_{\text{f}}^{\text{h}}-w_{\text{f}}^{\text{h}}\right)\cdot \nabla_{x}u_{\text{f}}^{\text{h}}\right)-\nabla_{x}\cdot \;\sigma_{\text{f}}(u_{\text{f}}^{\text{h}},p_{\text{f}}^{\text{h}}).$$

The definition of the parameters τ_MOM_, τ_CONT_ according to Pauli and Behr (2017) is given by

(47) $$\tau_{\text{MOM}}:\text{​​}=\min\left\{{\left(\frac{4}{\Delta t^{2}}+(u_{\text{f}}^{\text{h}}-w_{\text{f}}^{\text{h}})\cdot \text{G}(u_{\text{f}}^{\text{h}}-w_{\text{f}}^{\text{h}})\right)}^{-\frac{1}{2}},\frac{\rho_{\text{f}}C_{\text{M}}}{\mu_{\text{f}}\sqrt{\text{G}:\text{G}}}\right\},$$

with Δ*t* being the time step size and $\text{G}:={\frac{\partial \xi }{\partial \text{x}}}^{⊤}\text{K}\frac{\partial \xi }{\partial \text{x}}$, where $\frac{\partial \xi }{\partial \text{x}}$ denotes the Jacobian of the mapping from a physical FE to the reference FE, the tensor K is defined as

(48) $$\text{K}:\text{​​}=\frac{1}{2\sqrt[{3}]{2}}\left(\begin{array}{ccc} 3 & -1 & -1\\ -1 & 3 & -1\\ -1 & -1 & 3 \end{array}\right)$$

and the constant *C*_M_ = 0.0285. Further, the stabilization parameter τ_CONT_ is defined as

(49) $$\tau_{\text{CONT}}:\text{​​}=\frac{1}{\tau_{\text{MOM}}g_{\text{f}}\cdot \;g_{\text{f}}},$$

(50) $$g_{\text{f},i}:\text{​​}=\sum_{j=1}^{3}{\left(\frac{\partial \xi }{\partial x}\right)}_{ji}.$$

#### 2.3.3. Em-based kinematic driver model

Displacements computed with the EM model were used to prescribe the kinematics of the blood pool mesh which in turn was used for simulating hemodynamics in the CFD model. This was achieved by imposing ${\text{g}}_{\text{mov}}=\frac{\partial }{\partial t}{\text{d}}_{\text{s}}$ in Equation (38). Since the surface of the reference CFD blood pool mesh, $\partial \Omega_{\text{f}}^{0}$, is not conformal with the surface of the reference EM blood pool mesh, $\Omega_{\text{s},\text{bp}}^{0}$, and the overlap of the two surfaces is imperfect due to smoothing of $\partial \Omega_{\text{f}}^{0}$ and remeshing of $\Omega_{\text{f}}^{0}$, a direct transfer of displacements between the two surfaces is not readily feasible. As a remedy, we proceeded as follows. After solving the EM problem the subset of displacements ${\tilde{\text{d}}}_{\text{s}}$ that form the endocardial interface with the blood pool, $\Gamma_{\text{s},\text{bp}}^{0}$, were extracted from the solution **d**_s_ defined at $\Omega_{\text{s}}^{0}$. Since the mesh interface between $\Omega_{\text{s}}^{0}$ and $\Omega_{\text{s},\text{bp}}^{0}$ is conformal the extracted displacements can be applied as inhomogeneous time-varying Dirichlet boundary conditions to the blood pool mesh $\Omega_{\text{s},\text{bp}}^{0}$ to solve a linear elastic problem given as

(51) $$-\nabla_{X}\cdot \;\sigma (d_{\text{s}}(t))=\;0\text{ in }\Omega_{s,\text{bp}}^{0},$$

(52) $$d_{\text{s}}(t)=\;{\tilde{d}}_{\text{s}}(t)\text{ on }\partial \Omega_{s,\text{bp}}^{0},$$

where stress and strain tensor are

(53) $$\sigma (d_{\text{s}})\;:\text{​​}=\frac{E}{1+\nu }\left(\frac{\nu }{1-2\nu }\nabla_{X}\cdot \;d_{\text{s}}\;\text{I}+\;\varepsilon (d_{\text{s}})\right),$$

(54) $$\varepsilon (d_{\text{s}})\;:\text{​​}=\frac{1}{2}\left(\nabla_{X}d_{\text{s}}+{\left(\nabla_{X}d_{\text{s}}\right)}^{⊤}\right),$$

the constant *E* is Young's modulus in kPa and the constant ν is Poisson's ratio which is dimensionless in the range of [−1, 0.5). Combining the solutions **d**_s_ computed for $\Omega_{\text{s}}^{0}$ and $\Omega_{\text{s},\text{bp}}^{0}$ yields displacements **d**_s_ for $\Omega_{\text{s},\text{total}}^{0}$. Since $\partial \Omega_{\text{f}}^{0}$ is fully embedded in this domain, $\Omega_{\text{s},\text{total}}^{0}$ $\Omega_{\text{s},\text{total}}^{0}$ can be used as a hanging background mesh for interpolating displacements onto the blood pool mesh, $\Omega_{\text{f}}^{0}$, used for CFD simulations. However, for reasons of mesh quality, interpolation is solely applied on the boundary $\Omega_{\text{f}}^{0}$ itself, and to find the interior displacement field the exact same linear elastic problem 51–54, is solved for **d**_f_ instead of **d**_s_.

In both patient cases studied, ejection fractions were large leading to a substantial deformation of the blood pool mesh $\Omega_{\text{f}}^{t}$. To maintain mesh quality under such large deformations the parameters *E* and ν governing stiffness and incompressibility of the material were altered accordingly. Initially, a fixed *E*_0_ and ν_0_ was chosen while the subsequent modification of *E* and ν was guided by a combination of the two following strategies.

- *Quality based stiffening:* For each element τ_ℓ_ in the fluid mesh a tetrahedral quality indicator κ(τ_ℓ_) based on the movement from the previous time step was calculated, see Freitag and Knupp (2002) and Kanchi and Masud (2007), and rescaled such that for elements of good quality κ is close to 1, while for elements with poor quality κ tends toward infinity. Eventually, the parameter *E* was multiplied by κ within each element.
- ν*-Volume based stiffening:* For larger deformation elements in the fluid mesh may collapse or even invert, yielding a zero or negative volume. When solving Equations (51)–(54), the current element volumes were tracked and a volume ratio relative to an undeformed reference element was computed as $\frac{\left|\tau_{\ell }\right|}{\left|{\hat{\tau }}_{\ell }\right|}$. For ratios below a predefined critical value the parameter ν was set close to 0.5 to make this element nearly incompressible.

### 2.4. Numerical solution

Spatio-temporal discretization of all PDEs and the solution of the arising systems of equations relied upon the Cardiac Arrhythmia Research Package (CARP), see Vigmond et al. (2003). Numerical details on FE discretization (Rocha et al., 2011) and solution of EP (Vigmond et al., 2008b; Neic et al., 2012, 2017) and EM (Augustin et al., 2016b) have been discussed in detail elsewhere. FE discretization and solution of the Navier–Stokes equations were implemented recently using the same numerical framework which was extended to account for non-linear saddle-point problems arising from the discretized CFD equations.

Two time discretization schemes were implemented and compared for the applications in mind, and a computationally cheap semi-implicit scheme, modified from Forti (2016, section 1.4.2), showed similar results to the more expensive fully-implicit generalized-α method (Jansen et al., 2000). Hence, all results in section 3 were obtained using the semi-implicit scheme; to advance from time step *t*^*n*^ to *t*^*n*+1^, only a linear block system needs to be solved, where each block depends on data from the previous time step only. Solvers for the block system were taken from the PETSc library (Balay et al., 1997, 2016a,b). We used a right preconditoned flexible GMRES method with PETSc fieldsplit preconditioning (Silvester et al., 2001; Elman et al., 2008) which in turn uses BoomerAMG (Van Emden and Yang, 2002) to approximate sub-block inverses. While the time step size for mechanics and CFD was the same, Δ*t*_mech_ = Δ*t*_CFD_ = 0.5 ms, it was significantly smaller for EP, where Δ*t*_EP_ = 25 µ*s*.

The implementation of the CFD solvers has been subjected to various validation procedures against standard CFD benchmarks (Schäfer et al., 1996). All simulations were executed at the national HPC computing facility ARCHER in the United Kingdom using 384 and 768 cores for EM and CFD simulations, respectively.

### 2.5. Model parameterization

#### 2.5.1. Electrophysiology

Electrical activation sequences were indirectly parameterized using the QRS complex of a given patient's ECG as guidance. Unlike in previous studies (Augustin et al., 2016a), we refrained from a detailed parameterization which aimed at reproducing the QRS complex of the ECG for a given patient by finding appropriate locations and timings for the main fascicles of the cardiac conduction system in the LV. Rather, default locations and timings were used which yielded a total activation time within the physiological range.

#### 2.5.2. Passive biomechanics

The LV myocardium was characterized as a hyperelastic, nearly incompressible, transversely isotropic material with a nonlinear stress–strain relationship (Guccione et al., 1995). Orthotropic material axes were aligned with the local fiber, sheet and sheet normal directions. To remove rigid body motion, homogeneous displacement boundary conditions were applied by fixing the terminal rims of the clipped brachiocephalic, left common carotid and left subclavian arteries as well as the clipped rim of the aorta descendens, see Figure 1. The model was stabilized by resting the LV apex on an elastic cushion of which the bottom face was rigidly anchored also by applying homogeneous displacement boundary conditions.

The constitutive model was fitted to recorded clinical data as previously reported with minor modifications (Augustin et al., 2016a). The passive biomechanical model governed by the strain-energy function given in Equation (17) was fitted to approximate the end-diastolic pressure-volume relation (EDPVR). Due to limitations in the recorded data we refrained from directly fitting the model to the recorded pressure and volume data. Rather, only one data pair—EDV and end-diastolic pressure (EDP)—was used to fit the stress-free residual volume to the empiric Klotz relation (Klotz et al., 2007) by adjusting the isotropic scaling parameter *C*_Guc_ in Equation (17). As the model anatomy was built from a segmented 3DWH MRI scan—acquired during diastasis—the FE model was inflated to increase the volume of the cavity by the difference between the volume at mid diastasis and the EDV. Using the end-diastolic geometry, default material parameters and the recorded EDP, an initial guess of the stress-free reference configuration was computed by unloading the model using a backward displacement method (Sellier, 2011; Bols et al., 2013; Krishnamurthy et al., 2013). The unloading procedure was repeated with varying trial material parameters, *C*_Guc_, until the difference between the unstressed LV volume of the model and the prediction of the Klotz relation was less than 5 %.

#### 2.5.3. Active stresses

Parameters of the active stress model were fitted during IVC and ejection phase. During IVC the LV volume was held constant (Gurev et al., 2015) and the parameters of the active stress given in Equation (20) rate of contraction, τ_c_, and peak active stress, *S*_peak_, were manually adjusted to fit the maximum rate of rise of pressure, (d*P*/d*t*)_max_, and peak pressure, *p*_lv_.

#### 2.5.4. Afterload

**Figure 3:**
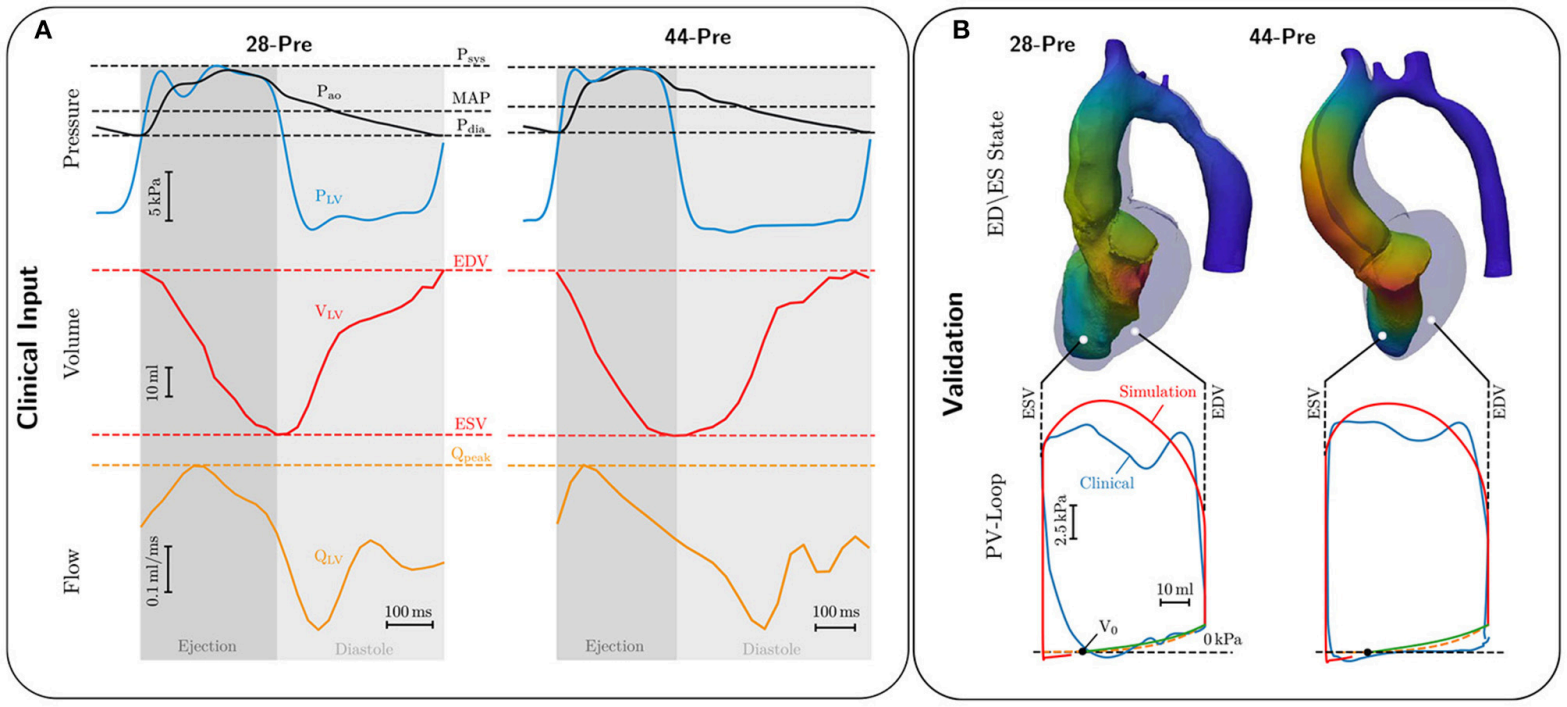
**(A)** Invasive clinical recordings from cases *28-Pre* and *44-Pre*. Top: Recorded aortic pressure P_ao_ (black curve) and recorded LV pressure *P*_LV_ (blue curve). Marked with dashed lines are Systolic pressure P_sys_, mean arterial pressure MAP, and diastolic pressure P_dia_; Center: Volume change in the LV, *V*_LV_, in red ranging from end-diastolic volume EDV to end-systolic volume ESV. Bottom: LV flow Q_LV_ in orange with marked peak flow Q_peak_. **(B)** Comparison of EM simulations and clinical data. Upper part shows a comparison of the LV model in end-diastolic (colored opaquley blue) and end-systolic configuration (colored by displacement). Lower part shows comparison of clinical (colored blue) and simulated PV loops (colored red). The dashed orange curve shows the ideal Klotz curve, while the green curve shows the simulated Klotz curve, with volume of stress-free unloaded configuration marked as *V*_0_.

When the LV pressure *p*_lv_ exceeded the aortic pressure, *p*_ao_, ejection was initiated by connecting the LV model with the lumped 3-element Windkessel model (Westerhof et al., 1971). Volume traces recorded from a given patient during ejection were used as input to compute aortic pressure traces by solving Equation (23). Both types of data were not recorded simultaneously as volume traces were computed from Cine MRI scans and pressure traces were recorded later invasively by catheterization. Volume and pressure traces were synchronized in time by aligning the onset of ejection of the volume trace *V*_lv_(*t*) with the instant of opening of the aortic valve in the pressure trace *p*_ao_(*t*). In those cases where heart rates were markedly different between the two measurements, volume traces were scaled in time to adjust LV ejection time (LVET) to the duration of ejection in the pressure traces, that is, the time elapsed between opening and closing of the aortic valve as these two instants in time were clearly identifiable in all traces *p*_ao_(*t*), see Figure 3. Moreover, volume traces were offset to ensure that the model volume based on the segmentation of the 3DWH scan acquired during diastasis matched up with the Cine-MRI based volume trace at mid diastasis. The parameter space of the Windkessel model comprising characteristic impedance of the aorta, *Z*_c_, as well as resistance, *R*, and compliance, *C*, of the arterial system was sampled using a recently developed stochastic sampling approach (Crozier et al., 2016b).

Numerous box constraints were used to constrain the search space of parameter sweeps. In particular, we used reported measurements in humans to define the mean values and restricted the search space for each parameter to fall within ± 20% around the mean. Due to high frequency errors introduced by the pressure transducer we refrained from computing norms ||*p*_ao, meas_ − *p*_ao, fit_|| to quantify the deviations of fitted from measured pressure and opted for manual selection using three criteria, aortic peak pressure, *p*_ao_, closing pressure of aortic valve and exponential decay of *p*_ao_ during diastole. For the sake of fitting *Z*_c_ we assumed *p*_ao_ ≈ *p*_lv_ since transvalvular pressure gradients in all patients were very minor.

#### 2.5.5. CFD boundary conditions

**Figure 4:**
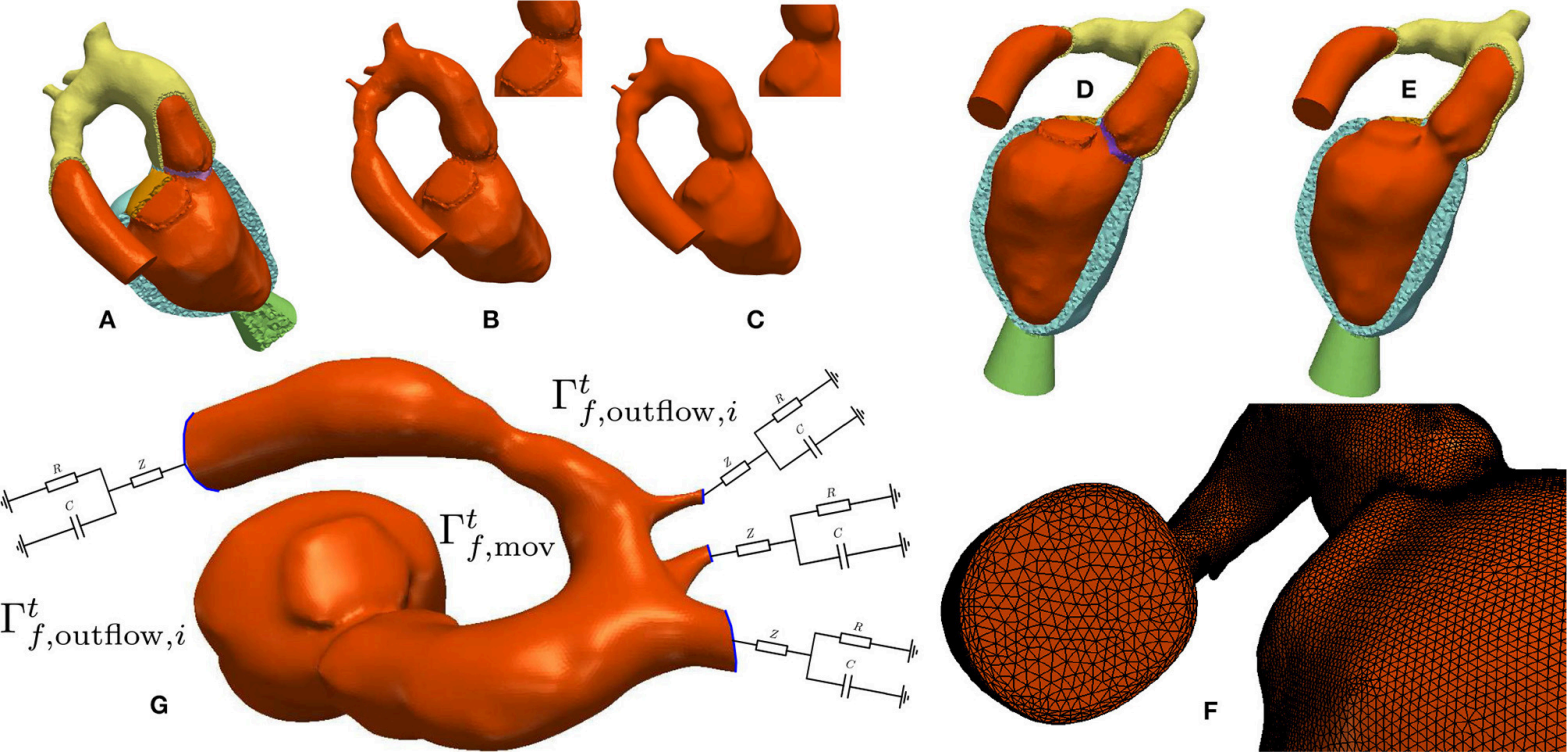
Processing workflow used for generating blood pool FE models: **(A,B)** Elements labeled as blood pool or valve were extracted from the mesh used for EM modeling. **(C)** Surfaces of extracted meshes were smoothed to avoid numerical instabilities due to reentrant corners resulting from a jagged surface. A closeup view of the smoothing effect is displayed in the upper right. The smoothed surface is then used as input for the fluid mesh generation. **(D,E)** Comparison of the smoothed and unsmoothed blood pool mesh immersed in the original EM mesh. **(F)** Closeup view of the generated boundary layer mesh. **(G)** Boundary conditions used for CFD. Moving wall boundary $\Gamma_{\text{f},\text{mov}}^{t}$ colored in orange, outlet boundaries $\Gamma_{\text{f},\text{outflow},i}^{t}$ colored in blue with attached illustration of the 3-element Windkessel models.

The validated EM models yield the time-dependent displacement fields, **d**_s_, which were transferred onto the fluid domain to drive simulations of blood flow in LV and aorta as described in section 2.3.3 yielding **d**_f_(*t*, **x**) defined on the whole CFD mesh. Figure 4G shows a summary of the boundary conditions. On the boundary $\Gamma_{\text{f},\text{mov}}^{t}$ a Dirichlet boundary condition enforcing the mesh velocity ${\text{w}}_{\text{f}}^{\text{h}}$ is applied. On each aortic outlet Γ_f, outflow, *i*_(*t*) a 3-Element Windkessel model as described in section 2.2.3 is attached. Further, the stabilization parameter β in Equation (39) was set to 0.2. Estimation of the input parameters for the hemodynamical Windkessel equations relied on an extension of the simple hydraulic analog of Ohm's law. Given the patient specific MAP, CO, and a percentage α_*i*_ of total CO running through the outlet the resistance *R*_*i*_ was estimated as

(55) $$R_{i}\approx \frac{\text{MAP}}{\alpha_{i}\text{CO}}.$$

The percentages α_*i*_ were obtained either by measurement or by applying Murray's law (Murray, 1926). The impedances *Z*_*i*_ were chosen as 5 % of *R*_*i*_, and the compliances *C*_*i*_ were chosen such that *R*_*i*_*C*_*i*_ ≈ 1, 000ms. To keep the semi-implicit character of the CFD system the Windkessel equations were solved with a semi-implicit backward Euler method using the flow $q_{i}^{n}$ through the aortic outlet, from the previous time step as input.

## 3. Results

### 3.1. Building electromechanical kinematic driver models

**Figure 5:**
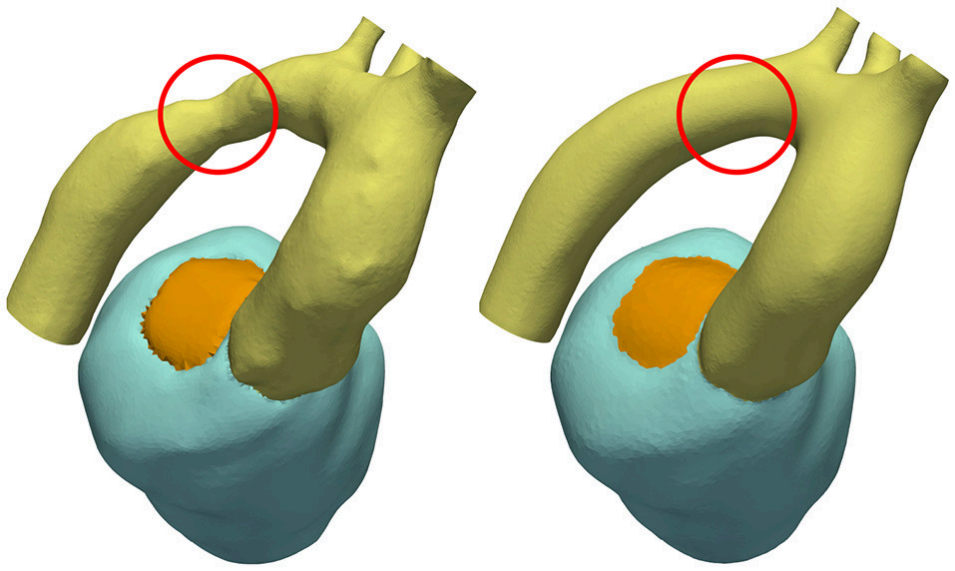
CoA anatomy of case 28 before and after virtual stenting procedure. CoA location is indicated with a red circle.

Using a previously developed automated workflow (Crozier et al., 2016a), anatomical FE models of LV and aorta were built for patient cases *28-Pre* and *44-Pre* based on segmented imaging data acquired under pre-treatment conditions. Figure 1 illustrates the key processing steps and the resulting FE model for case *28-Pre*. For the case *28-Pre* the CoA was repaired by a virtual dilatation procedure applied to the segmented image data with the aim to restore normal cross sectional areas. Subsequently, a new FE mesh was generated referred to as *28-Post*, which was essentially identical to *28-Pre*, with the only difference being the anatomical adjustment of the CoA in the aortic arch to the target post-treatment anatomy after stenting, see Figure 5.

Passive biomechanical properties, afterload and active stress models of cases *28-Pre* and *44-Pre* were parameterized using clinically recorded pressure and volume data under pre-treatment conditions, see Figure 3A. The fitted final parameters used are summarized in Table 2. The goodness of fit of both integrated EM models was verified by standard PV loop analysis as shown in Figure 3B. Results of a quantitative comparison with clinically derived metrics including EF, EDV and ESV, CO, and peak systolic pressure are summarized in Table 3.

**Table 3:** Comparison of clinical indicators and indicators computed from simulation for the EM models.

	**EM comparison**					
	**EDV_cl,sim_**	**ESV_cl,sim_**	**SV_cl,sim_**	**EF_cl,sim_**	**CO_cl,sim_**	**${\text{P}}_{\text{cl,sim}}^{\text{sys}}$**
	**(ml)**	**(ml)**	**(ml)**	**(%)**	**(ml/s)**	**(mmHg)**
*28-Pre*	88.16/87.47	30.62/31.02	57.54/57.14	65.27/64.81	87.46/86.85	146.037/139.362
*44-Pre*	91.68/91.67	31.59/30.95	60.10/60.72	65.54/66.24	76.31/76.32	158.413/135.236
rel. error [%]	0.78/0.01	1.3/2.0	0.69/1.03	0.70/1.07	0.69/0.013	4.57/14.63

### 3.2. Blood pool FE modeling for CFD

Conformal FE blood pool meshes were extracted from EM FE meshes, surfaces were smoothed and used for volumetric remeshing with increased spatial resolution including boundary layers. The corresponding workflow is illustrated in Figure 4.

**Figure 6:**
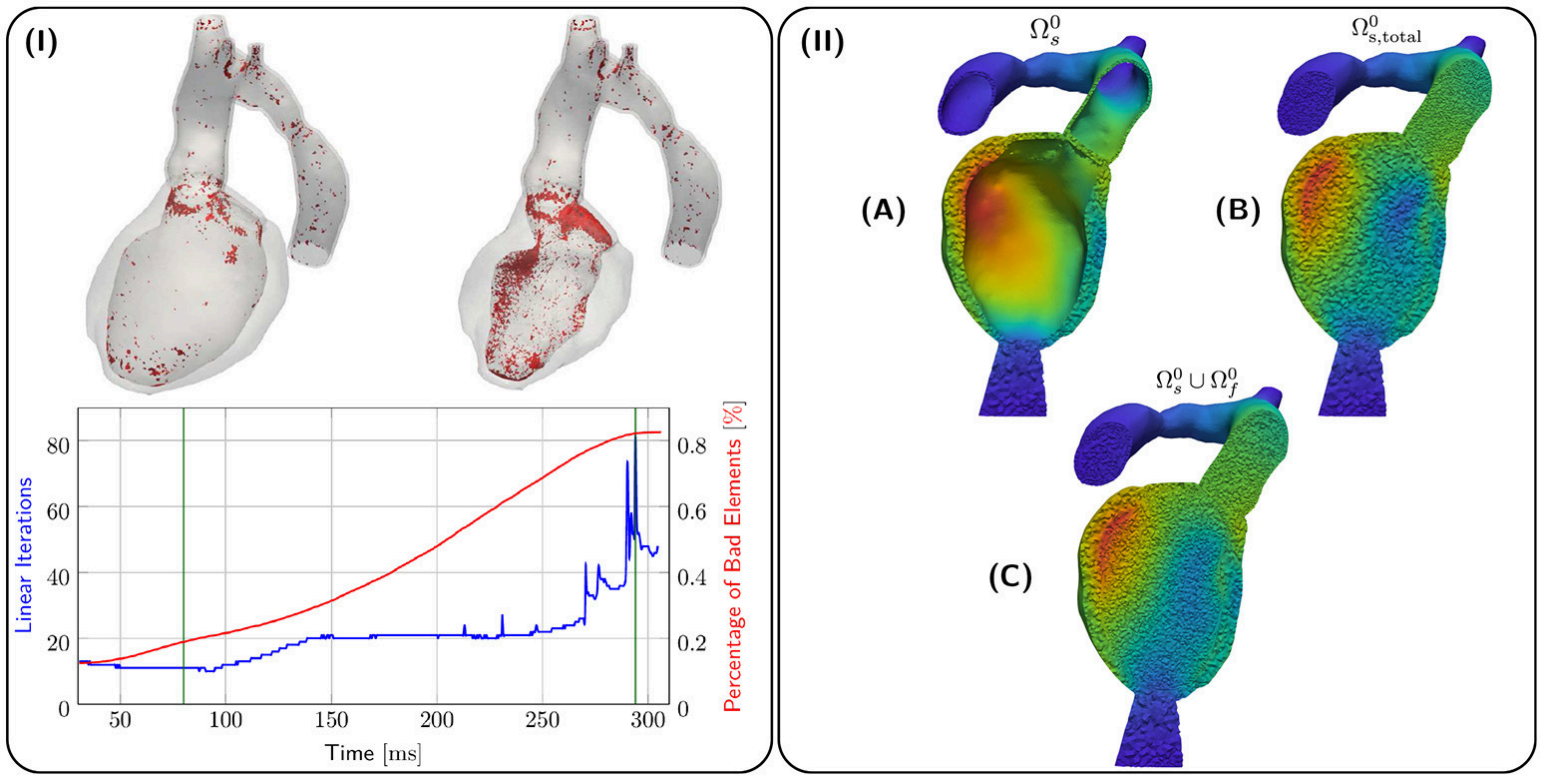
**(I)** shows quality analysis for case *28-Pre*. Spatial locations of elements of poor quality > 0.8 (in red) are shown at the top for different snapshots of deformation (green lines in graph). The graph below shows linear iterations per time step (in blue) and percentage of elements with poor quality > 0.8 (in red). **(II)** shows the processing stages of kinematic transfer for the *28-Pre* case at maximum displacement. **(A)** Displacement **d**_s_ on EM mesh $\Omega_{\text{s}}^{0}$. **(B)** Displacement **d**_s_ extended to conformal EM blood pool mesh $\Omega_{\text{s},\text{total}}^{0}$ which serves as hanging background mesh for the kinematic transfer onto the CFD blood pool mesh $\Omega_{\text{f}}^{0}$. **(C)** Displacement **d**_s_ on $\Omega_{\text{s}}^{0}$ superimposed with fluid mesh displacement **d**_f_ on $\Omega_{\text{f}}^{0}$.

Kinematics of the EM model were transferred to the CFD blood pool mesh and the result is illustrated in terms of displacements **d**_s_, **d**_f_ in Figure 6II. Due to the large EF of about 65 % for both *28-Pre* and *44-Pre*, the blood pool underwent a significant deformation. However, using a combination of element quality and ν-Volume based stiffening with an initial Young's Modulus *E*_0_ = 100 kPa and Poisson's ratio ν_0_ = 0.3, sufficient element quality was preserved throughout the entire ejection phase and numerical instabilities could be avoided. Figure 6I shows the 80th-percentile of bad element quality against the number of linear iterations required for convergence for the *28-Pre* case. The quality of elements was calculated with the same quality inidcator (Freitag and Knupp, 2002; Kanchi and Masud, 2007) as described in section 2.3.3 but was rescaled to the interval [0, 1], with the best element quality being 0 and the worst element quality being 1. The modest increase in iteration numbers of the iterative preconditioned GMRES solver provides indirect evidence of sufficiently preserved mesh quality (see Figure 6). Spatially, most lower quality elements were located in the CFD boundary layer.

### 3.3. Numerical CFD benchmarks

**Figure 7:**
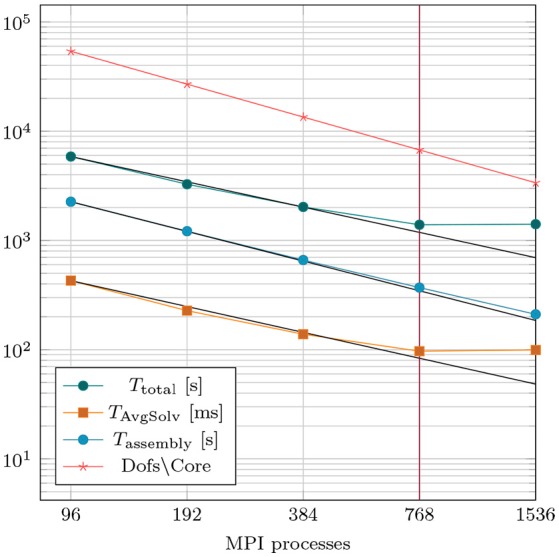
Results of strong scaling benchmark based on case *28-Post* with 5.2 million overall degrees of freedom. *T*_AvgSolv_ is the total solving time divided by the total amount of linear iterations per simulation run.

The implementation of the Navier–Stokes solver was verified by solving a set of standardized benchmark problems, see Schäfer et al. (1996). Computational performance was evaluated by performing strong scaling experiments by repeating the post-treatment hemodynamics simulation of case *28-Post* with varying numbers of cores ranging from 96 to 1.536. Details on computational complexity and costs are summarized in Table 4. For temporal discretization a time step of Δ*t* = 0.5 ms was used to simulate the ejection phase lasting for 208 ms. The overall discrete system comprised 5,177,056 degrees of freedom, which was solved over 416 time steps. Strong scaling results are summarized in Figure 7. Efficient strong scaling behavior was observed up to 768 cores with parallel efficiency slowly degrading from 100 % at 96 cores down to 55 % at 768 cores. Scalability stalled when doubling the core count to 1,536 which reduced the degrees of freedom per parallel partition down to 3,386. Parallel efficiency dropped to 27 % which is attributed due to the unfavorable ratio between local compute work and communication.

**Table 4:** Discretization details for the studied cases.

	**Electromechanics model**				**CFD model**				
	**NE**	**NV**	***h* [μm]**	**DOF**	**NE**	**NV**	***h* [μm]**	**DOFU**	**DOFP**
*28-Pre*	747,266	167,509	897	502,527	1,943,060	352,006	746.5	1,056,018	352,006
*28-Post*	632,635	149,174	954	447,522	7,405,128	1,294,264	531.6	3,882,792	1,294,264
*44-Pre*	727,194	168,804	997	506,412	2,285,005	412,728	717	1,238,184	412,728

*Shown are the number of elemens (NE), number of vertices (NV), average edge length h in μm, degrees of freedom for displacement (DOF), degrees of freedom for velocity (DOFU), degrees of freedom for pressure (DOFP)*.

### 3.4. Simulating cardiac and cardiovascular hemodynamics

**Figure 8:**
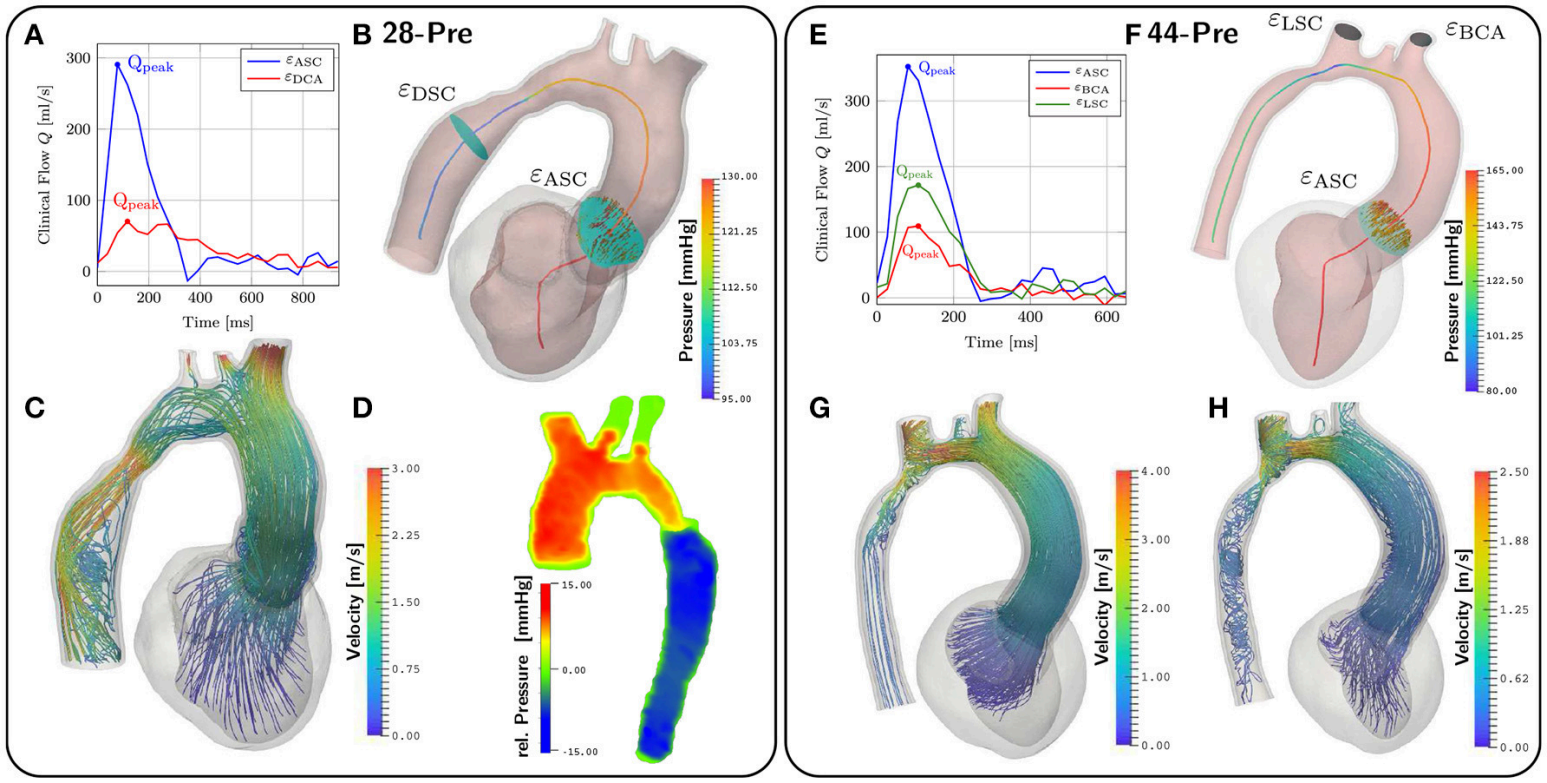
CFD results. **(A,E)** show the given clinical measurements for flow through different planes. The planes are depicted in **(B,F)**. **(B,F)** also depict the pressure along the centerlines at peak flow conditions at *t* = 167ms and *t* = 142ms respectively. **(C)** shows velocity streamlines at peak flow. **(D)** shows the relative pressure map from the Pressure–Poisson mapping used for validating the pressure drop in our simulations. **(G,H)** show velocity streamlines at peak flow and *t* = 200ms for case *44-Pre*.

Hemodynamics in the LV and aorta was simulated using the EM simulations as a kinematic driver. Flow rates through various aortic cross sections and outflow orifices were calculated as the integral over measured fluxes through the cross-sectional plane for both 4D VEC MRI and simulated flow data. At locations of interest which were ε_DSC_, ε_BCA_, ε_LCA_, and ε_LSCA_ denoting cross sections in the aorta descendens and the orifices of brachocephalic, left carotid and left subclavian artery, respectively, relative flows were computed from 4D VEC MRI data as fractions α_*i*_ expressed in percent of the total peak flow through the aorta ascendens as determined over the plane ε_ASC_. For those planes of interest where measurements were not feasible due to noise, flow percentages were estimated based on Murray's law. Flow curves during ejection at selected cross sections are shown in Figures 8A,E. MAP and computed mean flow through each outlet orifice were used to determine the parameters of the coupled Windkessel models of afterload in Equations (24, 25), see Table 5. In the *28-Pre* case this resulted in flow splits of α_*i*_ ≈ 23, 51.3, 12.83, and 12.83% whereas in the *44-Pre* case the flow split ratios were α_*i*_ ≈ 5.68, 57.45, and 34.01% for ε_DSC_, ε_BCA_, ε_LCA_, and ε_LSCA_, respectively.

**Table 5:** Windkessel parameters for each outlet of cases *28-Pre* and *44-Pre*.

	***28-Pre***				***44-Pre***			
	**DCA**	**BCA**	**RSC**	**LSC**	**DCA**	**BCA**	**RSC**	**LSC**
*R* [kPa ms/ml]	590.46	264.6	1, 058.24	1, 058.24	2, 480.07	276.01	466.23	7, 440.2
*Z* [kPa ms/ml]	29.52	13.23	52.91	52.91	124.003	13.9	23.31	372.01
*C* [ml/kPa]	1.69	3.78	0.944	0.944	0.403	3.62	2.14	0.134

For the CFD analysis a time step of Δ*t* = 0.5 ms was used. The ejection phases of the EM simulations were chosen as time horizons for the CFD simulation which lasted from *t* = 90 ms to *t* = 302 ms in the *28-Pre* case and from *t* = 70 ms to *t* = 329 ms in the *44-Pre* case, yielding 424 and 518 time steps, respectively. The Windkessel parameters for each outlet, calculated as described in section 2.5.5, are summarized in Table 5. Pressure *p*_f_ along the centerline *s*_c_ and fluxes through the planes ε_DSC_, ε_LSC_, ε_BCA_, and ε_ASC_ were computed at the instant of peak flow in the aorta ascendens and compared against measured data, which were pressures derived from Pressure–Poisson mapping (see Figure 8D) and 4D VEC MRI fluxes. For case *28-Pre* pressure drops were calculated from the pressure values on the intersection of the centerline and ε_DSC_, ε_ASC_ respectively. Further, we calculated the average pressure over the aforementioned planes as well. Both ways yielded a simulated pressure drop across the CoA of ≈ 29.2 mmHg which agreed well with the clinically estimated pressure drop of ≈ 30 mmHg. Furthermore, we calculated the flux through the various planes and compared them against the clinically estimated fluxes. A quantitative comparison of fluxes is given in Table 6. Figures 8C,G,H show velocity profiles at peak flow condtions. Figures 8B,F show the pressure along the centerlines, the velocity field ${\vec{v}}_{\text{f}}$ through the plane ε_ASC_, and the position of all planes used for evaluating fluxes. Supplementary Materials 1, 2 contain videos of the time evolution of the velocity distribution for cases *28-Pre* and *44-Pre*.

**Table 6:** Comparison of clincal estimated flow rates and simulated flow rates through the various planes for cases *28-Pre* and *44-Pre*.

**Flux comparison**						
		******28-Pre******		******44-Pre******		
	**Unit**	**ε_DCA_**	**ε_ASC_**	**ε_ASC_**	**ε_BCA_**	**ε_LSC_**
*Q*_peak, sim_	ml/s	85.5073	286.056	316.713	160.493	132.540
*Q*_peak, cl_	ml/s	70.3071	290.719	352.114	171.571	109.290
rel. error	%	21.62	1.604	10.054	6.46	21.27

### 3.5. Post-treatment simulations

**Figure 9:**
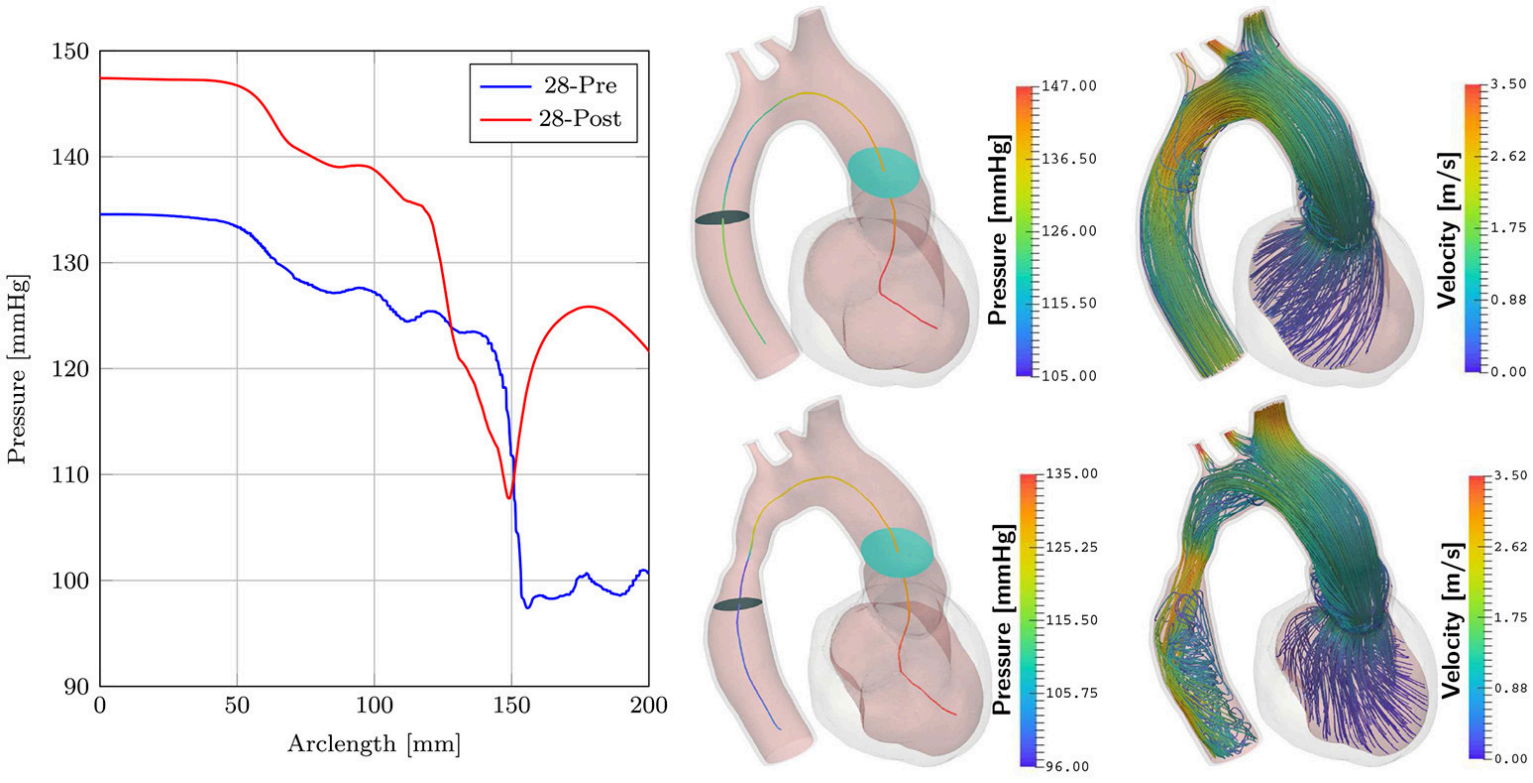
Comparison of cases *28-Pre* and *28-Post*. Shown on the left are the pressures along the centerline at peak flow. Depicted in the middle are the slices used for calculating the pressure drops. Shown on the right are velocity streamlines at peak flow.

Simulations of case *28-Pre* were repeated on geometry of case *28-Post* using almost the same set of parameters, see Table 2. Only *S*_peak_ was slightly adjusted, which resulted in a better peak pressure value in the LV. The geometry of case *28-Post* was almost identical to case *28-Pre* with the only exception being the virtual repair of CoA anatomy. In this scenario only pre- and post-treatment simulations were compared to evaluate their relative differences in terms of pressure and flow velocities. Figure 9 shows results. Pressure drops were calculated as in section 3.4 for both scenarios. For *28-Pre* we calculated a pressure drop of ≈ 29.2 mmHg while for *28-Post* a pressure drop of ≈ 14.15 mmHg was calculated.

## 4. Discussion

In this study, we report on the progress made toward a novel EMF model of the human LV that is entirely based on first principles and as such, in principle, is able to represent all cause-effect relationships with full biophysical detail. Unlike in the majority of cardiac CFD studies where the use of image-based kinematic driver models prevails, EM LV and aorta models of CoA patients were employed to serve as a kinematic driver to a computational model of hemodynamics in the LV cavity and aorta. A hybrid two stage modeling approach was adopted with regard to hemodynamics where EM and CFD model are executed sequentially. First, in the EM simulations the afterload imposed by the circulatory system upon the LV was represented by a lumped model to compute LV kinematics. These EM models were carefully fitted to available clinical data to replicate important clinical metrics characterizing hemodynamic and biomechanical work performed by the LV (Gsell et al., under review). In a subsequent step, a full-blown ALE-based CFD model with moving domain boundaries was *unidirectionally* or *weakly* coupled to the EM model. The motion of the fluid domain was driven by the kinematics of the EM model. Kinematics was transferred from EM mesh onto the CFD blood pool mesh by generating a combined kinematic model comprising LV, valve, aortic structure and a conformal blood pool mesh which served as a hanging background mesh for interpolation. The higher resolution blood pool CFD mesh with refined boundary layers was fully immersed in the EM background mesh. Kinematics was transferred by interpolation only onto the surface of the CFD blood pool mesh and extended into the volume of the blood pool by solving a linear solid mechanics problem.

We show validation results for two selected clinical CoA cases under pre-treatment conditions and compare between pre-treatment and post-treatment for one patient case in which the CoA was anatomically modified by a virtual stenting procedure. Further, we demonstrate numerical tractability of the implemented approach by providing strong scaling benchmark results. The overall cost of the entire work flow for building, fitting and execution of EMF simulations is comparable to plain image-based kinematic driver models (Mittal et al., 2016), suggesting that the proposed methodology may be, in principle, compatible with clinical time scales.

### 4.1. Biomechanical modeling vs. image-based kinematics

Modalities such as CMR and Cardiac CT on the other hand, provide excellent spatial resolution. CMR has an in-plane resolution of 1.5 × 1.5 mm, but more limited through-plane resolution (typically about 8 mm) while CT is capable of isotropic spatial resolution on the sub millimeter scale (≈ 0.5 mm) and clear delineation of trabeculae and lumen boundaries. CMR has the advantage of higher temporal resolution (30–50 ms) while temporal resolution in CT depends on the scanning system (50–200 ms). This is orders-of-magnitude lower than the temporal resolution required for the flow simulation (≈ 1, 000 phases per cardiac cycle) and appropriate interpolation methods need to be employed to create CFD-ready models. This stage of model generation has been very difficult to automate, and remains the biggest bottleneck for patient-specific cardiac flow modeling. Compared to pure image-based kinematic approaches our model is able to compute, e.g., the spatio-temporal distribution of wall stresses, power density, the length of diastolic intervals available for myocardial perfusion, O_2_ consumption, and metabolic supply/demand ratios. The variations of all these parameters in response to a changed afterload and many other biomarkers of physiological interest can be derived, which is not feasible with image-based models.

### 4.2. Kinematic transfer to CFD blood pool model

Both patients modeled in this study featured healthy EFs of > 60 %, that is, EF was ≈ 65 % in both cases. At a such high EFs the wall motion of the LV is significant, leading to substantial reductions in the LV blood pool volume. IB methods (Vigmond et al., 2008a; Seo and Mittal, 2013; Choi et al., 2015) are known to be more convenient to cope with the large deformation of the CFD blood pool (Quarteroni et al., 2017). IB methods and other non-boundary-fitting methods rely on a fixed fluid mesh and the moving wall of the ventricle is not explicitly tracked. The coupling between the CFD mesh and the structure is performed via Dirac Delta functions (IB) or Lagrange multipliers (fictitious domain methods) and is usually realized by introducing additional degrees of freedom on interface cut elements. While mesh generation is only necessary prior to computation fixed mesh methods typically require adaptive mesh refinement or modifications (Wang and Liu, 2004) to obtain reasonable accuracy for the solution near the fluid-solid interface.

In contrast, ALE algorithms capture the fluid-solid interface more accurately, are in general stable and easy to implement, no extra degrees of freedoms are introduced, and computational costs are low in comparison (Tallec and Mouro, 2001; van Loon et al., 2007). However, it is often assumed that unstructured FE approaches, as implemented in this paper, critically depend on automatic remeshing strategies (Long et al., 2013) to keep mesh quality within acceptable bounds (Mittal et al., 2016). Our study demonstrates that this may not necessarily be the case. While the mesh quality decreased with deformation over the course of ejection, the linear elastic deformation of the CFD blood pool mesh combined with the quality-based stiffening approach prevented the degeneration of any elements. The number of elements in which element quality degraded noticeably was very small. As illustrated in Figure 6, virtually all elements of reduced quality were located in the higher resolution boundary layer of the CFD blood pool mesh. According to the element quality metric used, an element quality of 1 refers to a fully degenerated element of zero volume. Despite the significant compression of the blood pool mesh, not a single element was deformed to this degree. Even when applying a stricter threshold where element quality is deemed poor if the quality indicator is > 0.8, which is not critical from a numerical point of view, the number of elements in this range remained small with < 0.8 % (Figure 6). The worst element quality observed in the entire mesh was 0.9994. Using a threshold of > 0.95 where element quality may be sufficiently poor to impact more notably on solver performance, only 24 out of 2,506,987 elements were found. Nonetheless, an increase in number of linear iterations required for convergence was observed which is likely to be linked to the gradual degradation of element quality. The number of iterations per solver step increased from around ≈ 17 iterations during early ejection up to ≈ 80 iterations during late ejection. While the more than fourfold increase in linear iterations negatively impacted overall solver performance and rendered simulations computationally more expensive, the complexity of automatic remeshing was avoided. We consider this a pivotal importance as automatic remeshing in combination with a MPI parallel FE solver is definitely feasible, but highly non-trivial to implement robustly and efficiently.

### 4.3. Computational feasibility

Computational feasibility of human scale cardiac simulations by using strongly scalable numerical implementations has been demonstrated previously for electrophysiology (Niederer S. et al., 2011) and mechanics (Augustin et al., 2016b). More recently, we reported on a novel reaction-eikonal model which reduces the cost of EM simulations significantly by alleviating constraints imposed by reaction-diffusion models upon mesh resolution (Neic et al., 2017). In this study, this recent reaction-eikonal approach was used for simulating EM using the same FE grid with an average resolution of ≈ 1 mm for both EP and mechanics. Such lower resolutions suffice for solving for mechanics with sufficient accuracy (Land et al., 2015). The overall reduction in terms of nodes and degrees of freedom reduces the compute cost substantially, rendering simulations in desktop environments feasible. Using 96 cores, EM simulations of a full cardiac cycle only lasted ≈ 180 min which facilitated sufficiently short simulation cycles for efficient model fitting. The entire workflow for building and parameterizing one patient-specific EM model is feasible within a day.

Owing to the higher resolution of the blood pool mesh and the presences of a refined boundary layer the number of nodes and degrees of freedom were higher than for EM simulations, around 350,000/1,500,000 nodes/degrees of freedom for case *28-Pre* and 400,000/1,700,000 nodes/degrees of freedom for case *44-Pre*, respectively. To assess strong scaling properties of our CFD solver implementation, the resolution was further increased to 1,300,000/5,000,000 nodes/degrees of freedom for case *28-Post* to cover a wider range of core counts. Strong scaling efficiency leveled off when doubling from 768 to 1,536 cores. Local compute load with 1,536 was 900/2,600 nodes/degrees of freedom per core. The patient simulations were performed using 384 cores, resulting in a load per core of about 900/2,700 nodes/dofs, respectively. At these resolutions CFD simulations were executed in ≈ 40 min, suggesting that compatibility with clinical time frames will be achievable.

### 4.4. Limitations

In the presented modeling approach numerous simplifying assumptions were made which may affect the biophysical fidelity of the model. In particular, while the aorta was taken into account as a solid structure in the EM simulations, its biomechanical description was simplified by assuming isotropic behavior, that is, the fibrous organization of aortic walls remained unaccounted for (Augustin et al., 2014). Further, as our main focus was on the EM of the LV and, to a much lesser degree, on the aorta, the aortic lumen remained unpressurized and, in absence of distensibility measurements of the aortic wall, parameters of the passive biomechanics model used for the aortic wall were not fitted. Thus the model of the aorta does not respond to the rise in pressure during ejection with an adequate distension Δ*V* of its lumen. In the CFD simulations Δ*V* ≈ 0 translates into a stiff aorta of low compliance which may cause a bias toward overestimation of the computed pressure fields. Further, the influence of the aortic valve upon blood flow was not taken into account. Rather, it was assumed that with the start of ejection the aortic valve is in its full open configuration, which allows blood flow over the entire orifice area and in which the valve does not influence the blood flow out of the LV in a significant way. Since only CoA patients were modeled which showed no indications of AVD this simplifying assumption may be well justified.

A potential main strength of the presented modeling approach—the ability to predict the biomechanical response of the LV to changed flow patterns in the aorta—was not exploited. Due to the weak FSI coupling the immediate feedback of altered flow or changed pressure gradients in the aorta on LV biomechanics was ignored. In our current modeling approach any such feedback must be mediated through changes in the parameterization of the lumped afterload model. However, owing to regulatory mechanism of the circulatory system level this is not directly predictable with the modeling setup used in this study as flow distribution through the four outlets will be influenced by factors which cannot be accounted for in a model comprising only LV, aorta and lumped outflow impedances. In any case, one cannot assume that the computed changes in pressure gradients across a CoA translate directly into a reduction in LV peak pressure. Independently of the modeling approach taken—be it a strongly or weakly coupled FSI model—a lumped model of systemic regulation is likely to be necessary to predict altered LV loading under post-treatment conditions (Arts et al., 2005; Lumens et al., 2009). Compared to a fully coupled FSI model our approach is limited in the sense that CFD simulations do not influence the behavior of the EM model. However, in many clinical settings CFD simulations in the aortic arch and LV with image based kinematics prevail.

Image based kinematic models can only depict the *status quo* of a patient. With our personalized EM model, based on first principles, we can do simulations altering the motion, simply by changing input paramters. The altered motion is then reflected in the CFD simulation. Examples would include changes in heart beats, infarcts or LBBB conditions.

In this work, the effect of stenting was only accounted for by a geometric change in the computational geometry and an *ad hoc* adjustement of the lumped model parameters. In future studies, we intend to use a 1-D model of the arterial tree coupled to a 0-D lumped model at the aortic outlets, thus being able to account for the effect of stenting in a more detailed fashion, see for example Quarteroni et al. (2017). As a first step toward our ultimate goal of a fully coupled FSI model, that is based entirely on first principles, we will add the dynamic fluid pressure $\frac{\rho_{\text{f}}}{2}{\left|{\text{u}}_{\text{f}}\right|}^{2}$ to the pressure of the lumped model (0-D or 1-D). This results in a spatio-temporal pressure inside the LV and the aorta, and to incorporate the dynamic feedback of fluid upon structure we will iterate between a CFD solving step and a EM solving step within each timestep to guarantee a converged solution.

## 5. Conclusion

Biophysically detailed models of LV EM can be efficiently built and parameterized with clinical data to be considered a viable option for patient-specific simulation. Similar to image-based kinematic models such biophysics-based EM models can be used as a kinematic driver for simulating cardiac and vascular hemodynamics. The cost of model building and execution is comparable between the two approaches. Biophysical EM models offer the significant advantage of being based entirely on first principles and as such, may allow to make predictions of interventions altering pressure and flow patterns onto LV performance. In contrast, image-based kinematics modeling may provide a more accurate representation of blood pool motion, at least under pre-treatment conditions or post-treatment conditions secondary to interventions which do not influence LV kinematics in a significant way.

## Author contributions

EK, GP contributed conception and design of the study; LG, TK acquired and processed clinical data; EK, MG, AN, and CA developed numerical methodology; AP contributed by conceiving modeling workflows and FE meshing; LM and MG developed parameterization of electromechanical model; EK, MG, CA, and GP analyzed and interpreted simulation data; EK, CA, and GP drafted the article; EK, CA, MG, LG, and GP critically revised the article; All authors contributed to manuscript revision, read, and approved the submitted version.

## Funding

This research was supported by the grants F3210-N18 and I2760-B30 from the Austrian Science Fund (FWF), the EU grant CardioProof agreement 611232 and a BioTechMed award to GP, and a Marie Skłodowska–Curie fellowship (GA 750835) to CA. We acknowledge PRACE for awarding us access to resource ARCHER based in the UK at EPCC (grant CAMEL) and the Vienna Scientific Cluster VSC-3.

### Conflict of interest statement

The authors declare that the research was conducted in the absence of any commercial or financial relationships that could be construed as a potential conflict of interest.

## Footnotes

^1^http://www.gyrotools.com/products/gt-flow.html

^2^https://www.mevis.fraunhofer.de/en/solutionpages/mevisflow-non-invasive-interactive-exploration-of-in-vivo-hemodynamics.html

^3^https://amira.zib.de

^4^http://www.cgal.org

^5^http://www.vmtk.org

^6^http://www.ansys.com/Services/training-center/platform/introduction-to-ansys-icem-cfd-Hexa
